# Disease-associated mutations impacting BC-loop flexibility trigger long-range transthyretin tetramer destabilization and aggregation

**DOI:** 10.1016/j.jbc.2021.101039

**Published:** 2021-07-31

**Authors:** Sebastián A. Esperante, Nathalia Varejāo, Francisca Pinheiro, Ricardo Sant'Anna, Juan Román Luque-Ortega, Carlos Alfonso, Valentina Sora, Elena Papaleo, Germán Rivas, David Reverter, Salvador Ventura

**Affiliations:** 1 Institut de Biotecnologia i de Biomedicina and Departament de Bioquímica i de Biologia Molecular, Universitat Autònoma de Barcelona, Bellaterra, Barcelona, Spain; 2 Molecular Interactions Facility, Centro de Investigaciones Biológicas Margarita Salas, CSIC, Madrid, Spain; 3 Systems Biochemistry of Bacterial Division Laboratory, Centro de Investigaciones Biológicas Margarita Salas, CSIC, Madrid, Spain; 4 Computational Biology Laboratory, Danish Cancer Society Research Center, Copenhagen, Denmark; 5 Cancer Systems Biology, Health and Technology Department, Section for Bioinformatics, Technical University of Denmark, Lyngby, Denmark

**Keywords:** transthyretin, amyloid, aggregation, protein structure, protein stability, molecular dynamics, ERAD, endoplasmic reticulum-associated degradation, FAC, familial amyloid cardiomyopathy, FAP, familial amyloid polyneuropathy, MD, molecular dynamics, RMSD, root mean square deviation, RMSF, root mean square fluctuation, TEM, transmission electron microscopy, TTR, transthyretin, WT, wild-type

## Abstract

Hereditary transthyretin amyloidosis (ATTR) is an autosomal dominant disease characterized by the extracellular deposition of the transport protein transthyretin (TTR) as amyloid fibrils. Despite the progress achieved in recent years, understanding why different TTR residue substitutions lead to different clinical manifestations remains elusive. Here, we studied the molecular basis of disease-causing missense mutations affecting residues R34 and K35. R34G and K35T variants cause vitreous amyloidosis, whereas R34T and K35N mutations result in amyloid polyneuropathy and restrictive cardiomyopathy. All variants are more sensitive to pH-induced dissociation and amyloid formation than the wild-type (WT)-TTR counterpart, specifically in the variants deposited in the eyes amyloid formation occurs close to physiological pHs. Chemical denaturation experiments indicate that all the mutants are less stable than WT-TTR, with the vitreous amyloidosis variants, R34G and K35T, being highly destabilized. Sequence-induced stabilization of the dimer–dimer interface with T119M rendered tetramers containing R34G or K35T mutations resistant to pH-induced aggregation. Because R34 and K35 are among the residues more distant to the TTR interface, their impact in this region is therefore theorized to occur at long range. The crystal structures of double mutants, R34G/T119M and K35T/T119M, together with molecular dynamics simulations indicate that their strong destabilizing effect is initiated locally at the BC loop, increasing its flexibility in a mutation-dependent manner. Overall, the present findings help us to understand the sequence-dynamic-structural mechanistic details of TTR amyloid aggregation triggered by R34 and K35 variants and to link the degree of mutation-induced conformational flexibility to protein aggregation propensity.

Amyloid diseases constitute a heterogeneous group of clinical disorders characterized by protein misfolding, aggregation, and the deposition of insoluble fibrils in a variety of tissues and organs (1, 2). Although amyloidogenic proteins are derived from different and unrelated sources, they all converge to form similar structures made by cross-β sheets, termed amyloid fibrils. Amyloid deposition can be confined to a particular organ or tissue, leading to localized amyloidosis (*e.g.*, Alzheimer's disease, prion diseases, type 2 diabetes mellitus). Conversely, protein deposition can also occur systemically throughout the body, leading to systemic amyloidosis (*e.g.*, amyloid light chain (AL), amyloid-A (AA), and hemodialysis-associated (Aβ2m), amyloidosis) (3).

Transthyretin (TTR) is one of more than 30 amyloidogenic proteins associated with amyloid diseases (4). TTR is an extracellular soluble nonglycosylated tetrameric protein synthesized mainly in the liver, retinal pigment epithelium, pancreas, and choroid plexus. TTR transports holoretinol-binding protein and is a minor carrier of thyroxine (T_4_) in the blood, whereas in the cerebrospinal fluid, it is the primary carrier of T_4_ (5). Extracellular misfolding and misassembly of TTR lead to the formation and accumulation of amyloid fibrils in a variety of tissues, which gives rise to distinct progressive clinical syndromes known as TTR-related amyloidosis. The propensity of TTR to form amyloids is associated with aging and/or mutations that destabilize the native state. Deposition of wild-type (WT) TTR within the extracellular matrix of the heart and other tissues causes senile systemic amyloidosis (SSA), a late-onset sporadic cardiomyopathy, affecting as much as 25% of the population over 80 years of age (6, 7).

More than 130 mutations within the TTR gene have been associated with autosomal dominant familial forms of amyloidosis, which typically present earlier onset and are often severe (8). Most of the pathogenic variants identified so far correspond to missense mutations and display tissue-selective amyloid deposition and pathology. Familial amyloid polyneuropathy (FAP) and familial amyloid cardiomyopathy (FAC) are the most common forms of hereditary amyloidosis, affecting the peripheral nervous system and the heart, respectively. The V30M variant is the most common mutation associated with FAP (9), whereas V122I and I68L are predominantly associated with FAC (10, 11). Highly destabilizing mutations, such as A25T (12) and D18G (13), cause selective amyloidosis restricted to the central nervous system. These variants are synthesized in the liver, but the misfolded proteins are retained in the endoplasmic reticulum and degraded intracellularly by the endoplasmic reticulum-associated degradation (ERAD) mechanism (14). Contrarily, when the synthesis occurs in the choroid plexus due to the high availability of T_4_, this small molecule acts as a metabolite chaperone, stabilizing the tetramer, which evades the proteostasis ERAD mechanism. Once secreted, dissociation of T_4_ promotes the destabilized TTR tetramer to dissociate, misfold, and aggregate.

Even though most of the mutations destabilize the TTR tetramer and favor amyloid formation, some disease protective mutations have also been identified. Heterozygotes harboring V30M and T119M mutations were protected from developing FAP (15). The presence of T119M in the heterotetramers reduces its dissociation rate and, consequently, the aggregation rate and amyloidogenesis progression. This mechanism, known as kinetic stabilization, laid the foundation for therapeutic strategies to ameliorate TTR amyloidosis (16). Several small molecules that bind and kinetically stabilize the nonamyloidogenic tetramer have been identified. For instance, the benzoxazole tafamidis (17) has shown safety and efficacy in slowing disease progression of polyneuropathy (18) and cardiomyopathy (19), and it was approved by different regulatory agencies worldwide. Tolcapone is another promising potent kinetic stabilizer of TTR that crosses the blood–brain barrier and might find therapeutic application in CNS amyloidosis (9, 11, 20).

The sequence-dependent factors that lead to TTR aggregation have been extensively studied over the last 25 years (14, 21, 22). WT-TTR homotetramers or heterotetramers comprised of mutant and WT subunits dissociate into monomers, which can rapidly misfold and subsequently self-assemble into amyloid fibrils. It is well established that TTR tetramer dissociation is the rate-limiting step for amyloid fibril formation. In this scenario, pathogenic mutations can destabilize the tetramer, decreasing intersubunit affinity and/or increasing the dissociation rate. Once the tetramer is dissociated, mutations can influence the monomer thermodynamic stability, thus increasing the population of the aggregation-prone misfolded monomer. However, we still do not understand why a mutation in a given residue and especially why different substitutions of the same residue may lead to different clinical manifestations. This is exemplified by the R34G, R34T, K35N, and K35T mutations identified in patients with familial amyloidosis. R34 and K35 are two positively charged residues residing at the C-terminus of TTR β-strand B. We have shown that, *in vitro*, these sequentially adjacent positive charges contribute to protect TTR against aggregation (23), which may justify why the loss of one of these charges results in amyloidosis. However, this generic mechanism does not explain why the R34G- and K35T-TTR variants were identified in patients with vitreous amyloidosis (24, 25), whereas R34T and K35N mutations were described in families with amyloid polyneuropathy and restrictive cardiomyopathy (26, 27).

In the present study, we investigate the pathogenic mechanism of missense mutations affecting residues R34 and K35 of TTR, using computational, biophysical, and structural approaches. The results contribute to understand why different mutations of the same TTR amino acid and the same substitution in adjacent residues in a given structural element might elicit different disease manifestations.

## Results

### pH-induced amyloidogenicity of missense mutations at residues 34 and 35 of TTR

**Figure 1:**
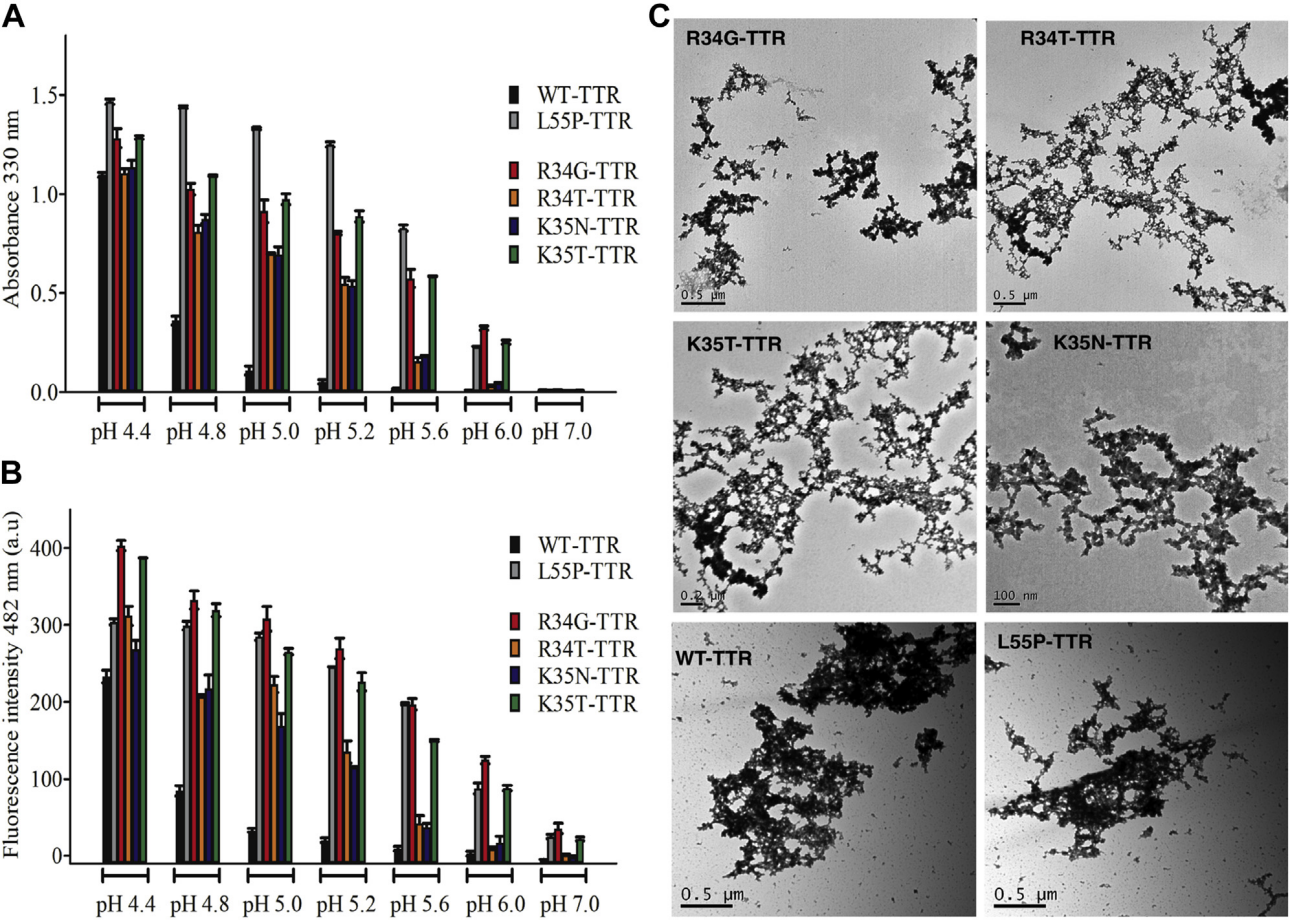
**pH dependence on aggregation propensity and amyloid protofibril formation of TTR disease associated variants.** 3.5 μM of WT and pathogenic TTR variants were incubated in 100 mM Tris.HCl, 50 mM MES, 50 mM sodium acetate, and 0.1 M KCl at different pHs (ranging from 4.4 to 7.0), for 72 h at 37 °C prior measurements. The amount of *protofibril* formation as a function of pH was determined by measuring the suspension turbidity at 330 nm (*A*) and the increase in fluorescence intensity at 482 nm in ThT-binding assays (*B*). Each experiment was performed in triplicates. *C*, TEM images of the aggregates of TTR variants, formed at pH 4.4 at 37 °C. Scale bars values are indicated.

WT-TTR is converted into amyloid fibrils by pH-mediated tetramer dissociation. The dissociation is linked to tertiary structural changes resulting in the formation of a monomeric amyloidogenic intermediate. The optimal pH to induce WT-TTR tetramer dissociation and the concomitant monomeric structural rearrangement is pH 4.4, whereas at pHs closer to physiological values, WT-TTR is unable to form amyloid fibrils (21). We started investigating the effect of missense mutations involving residues R34 and K35 of TTR on pH-induced amyloidogenicity. The dependence of aggregation and amyloid formation of TTR variants on pH was monitored by measuring turbidity at 330 nm and ThT binding, respectively (Fig. 1, *A* and *B*) and compared with those of WT-TTR and the L55P-TTR mutant (28). Both aggregation and amyloid formation increase, as we move from pH 7.0 to pH 4.4 in all the studied variants. Surprisingly, at pH 5.6 and 6.0, the R34G- and K35T-TTR mutants displayed similar turbidity and ThT-binding ability than that of L55P-TTR, the most pathogenic TTR mutant described so far. It should be noted that at pH 7.0, all TTR variants exhibited low turbidity, but R34G-, K35T-, and L55P-TTR displayed a 20-fold increase in ThT-binding fluorescence intensity compared with WT-TTR. This finding suggests some extent of detectable amyloid formation at neutral pH, as previously described for L55P-TTR (28). The morphological properties of the aggregates formed upon incubation at pH 4.4 were further evaluated by transmission electron microscopy (TEM) that confirmed the presence of protofibrilar aggregates in all cases (Fig. 1*C*). Thus, the positively charged residues at positions 34 and 35 are critical for preserving the native, nonaggregation prone conformation of TTR. Strikingly, it is an interplay between the mutated position and the substitution type, which seems to determine the TTR variant aggregation behavior.

### Conformational stability of TTR variants

**Figure 2:**
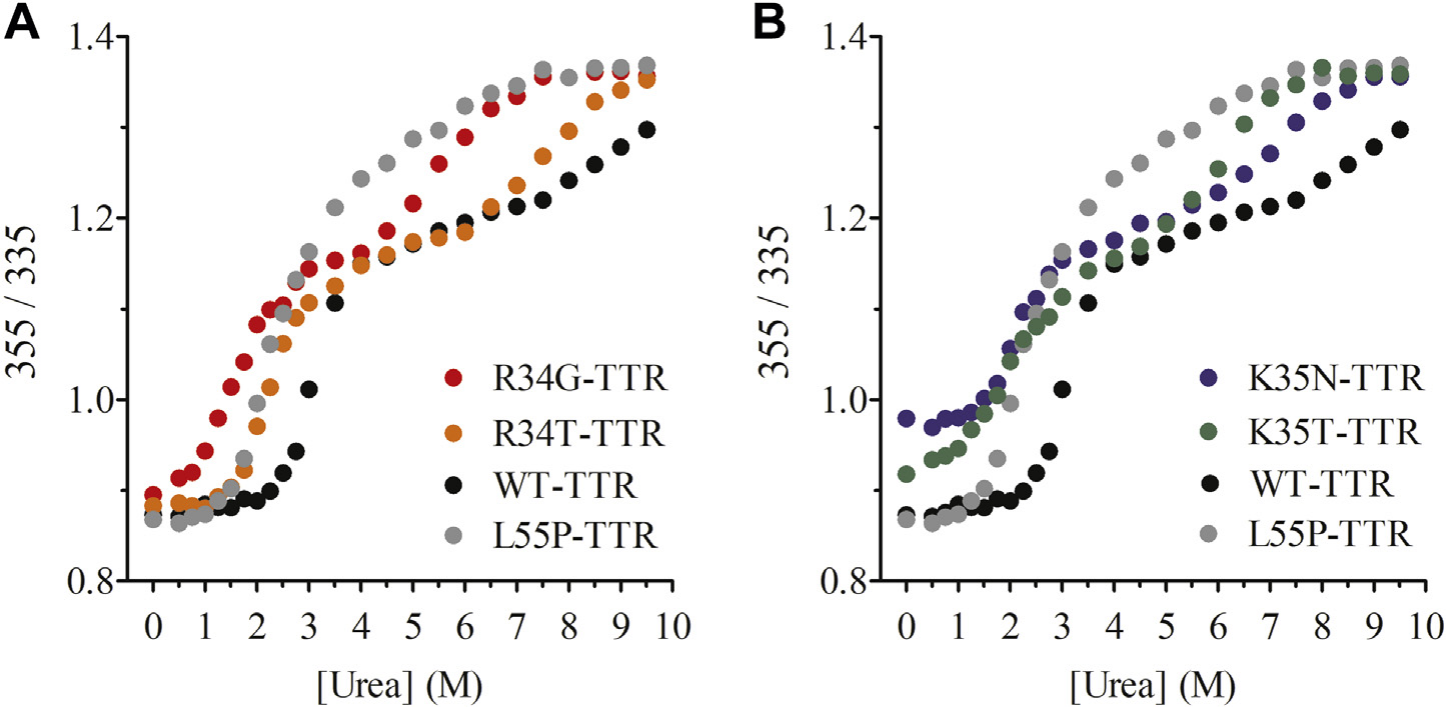
**Urea equilibrium denaturation of TTR disease associated variants.**1.5 μM of WT- and pathogenic TTR variants were incubated for 96 h at 25 °C in 50 mM sodium phosphate (pH 7.4) containing 0.1 M KCl with increasing urea concentrations prior measurements of tryptophan fluorescence emission spectra. The tryptophan fluorescence emission intensity ratio displayed is defined as the ratio of the tryptophan emission intensity at 355 nm (unfolded state) to the tryptophan emission intensity at 335 nm (folded state) and is used as a measure of foldedness as was previously described (22). The missense variants in residues 34 (*A*) and 35 (*B*) are plotted in the same graph with the denaturation curves of WT-TTR and L55P TTR to compare the denaturation mechanism.

TTR pathogenic mutations reported so far affect either tetramer stability or thermodynamics of misfolding of monomeric TTR. To understand the pathogenic mechanism of missense mutations involving residues 34 and 35, we investigated the effects of these variants on TTR tertiary and quaternary structural stabilities. We probed the tertiary structure's conformational stability by urea denaturation experiments monitored by tryptophan fluorescence spectroscopy (Fig. 2). Tryptophan fluorescence emission spectra of TTR variants in native conditions revealed that the emission maximum wavelength of K35T, and especially K35N, was red-shifted relative to R34 variants and WT-TTR, suggesting a distinct tryptophan environment (Fig. S1). The four studied variants, together with WT- and L55P-TTRs, were analyzed to compare their dissociation/unfolding mechanisms. It is important to note that after 96 h incubation, WT-TTR did not reach a stable unfolded state baseline at high urea concentrations, indicating that the protein was not fully unfolded. Completely unfolding was achieved using Gdm.HCl as denaturant (Fig. S2). Remarkably, the 355/335 ratio and the fluorescence center of spectral mass value of the Gdm.HCl denaturation midpoint coincide with that of ∼5.0 M urea (Fig. S2). This finding suggests that, in our assay conditions, after 96 h incubation in 5.0 M urea, ∼50% of WT-TTR remains tetrameric. This urea denaturation biphasic conformational behavior was previously reported (29) and attributed to a fraction of anion-stabilized tetrameric TTR, which is highly resistant to urea denaturation. Thus, two types of TTR tetramer populations likely coexist, the nonstabilized tetramers, which dissociate and unfold with a transition midpoint C_m1_ = 3.2 M (at 1.5 μM protein concentration), and stabilized tetramers, which cannot be completely unfolded on the experimental timescale.

The R34-TTR variants exhibit two separated transitions, the first one with an estimated midpoint of C_m1_ = 1.7 M for R34G and C_m1_ = 2.2 M for R34T. At the urea concentration range from 4.0 to 6.0 M, the 355/335 intensity signal stabilizes, while above 6.0 M, a second transition is observed with midpoints of C_m2_ = 5.0 M and C_m2_ = 7.0 M for R34G and R34T, respectively (Fig. 2). Noticeably, the first transition of R34T is coincident with the single broad transition displayed by the L55P-TTR mutant. Altogether, the specific residue at position 34 affects both transitions differently, with the glycine residue exerting the largest destabilization effect, as evidenced by the shift of both transition midpoints to lower urea concentrations.

The urea denaturation curves of the missense mutants at residue K35 also displayed two transitions, the first one with an estimated C_m1_ of 2.2 M for K35N and 2.1 M for K35T, and a second transition midpoint with C_m2_ = 6.0 M for K35T and C_m2_ = 7.0 M for K35N. The urea denaturation curves for R34 and K35 mutants suggest a similar dissociation/unfolding mechanism. Compared with WT-TTR, both transitions are shifted to lower urea concentrations, suggesting destabilization of both tetramer populations mentioned above.

Urea denaturation curves of TTR variants at increasing protein concentrations revealed an increase in the first transition midpoint as the protein concentration increases, indicative of a dissociation process. The second transitions were nearly coincident, irrespective of the protein concentration (Fig. S3).

Resveratrol binding is an ideal probe to assess TTR quaternary structural changes, as the tetramer can bind resveratrol, whereas the monomer cannot (22). Quaternary structural changes induced by increasing urea concentration were monitored spectroscopically by measuring resveratrol fluorescence. Accordingly, two transitions were also observed, overlapping with the tryptophan fluorescence center of spectral mass (Fig. S4). Resveratrol-binding estimates of the tetramer percentage as a function of urea concentration reveal that the first posttransition baseline corresponds to 30–50% of tetramers (Fig. S4).

To better understand the chemical denaturation mechanism, equilibrium denaturation experiments were also performed using Gdm.HCl as a denaturant. All TTR variants showed a single transition, displaying an estimated C_m_ = 3.5 M for both R34G and K35T, a C_m_ = 4.5 for K35N and C_m_ = 4.7 for R34T-TTR (Fig. S5). WT-TTR also displayed a single transition with a C_m_ = 5.1 M Gdm.HCl, indicating that the variants destabilized the tetramer to different extents. Size-exclusion chromatography performed at 2.0 M Gdm.HCl revealed that all the variants remain tetrameric in the pretransition region (Fig. S5), suggesting a simple two-state mechanism (tetramer to unfolded monomer) or a three-state mechanism with a single transition (tetramer dissociation to monomer and monomer unfolding linked).

Importantly, independent of the employed denaturant and probe, the more amyloidogenic R34G and K35T TTR tetramers turned to be less stable than their R34T and R35N counterparts, all the mutants being destabilized relative to WT-TTR.

### Quaternary structure of TTR urea denaturation intermediates

**Figure 3:**
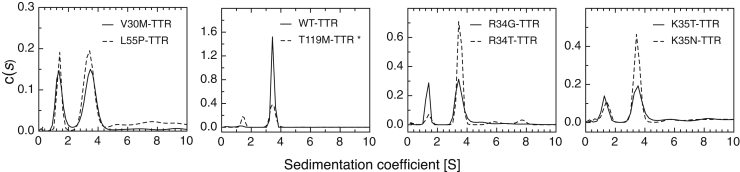
**Quaternary structure of urea denaturation intermediates of TTR variants.** Sedimentation coefficient distribution of TTR variants determined by a sedimentation velocity experiment. TTR variants at 5.0 μM were incubated for 96 h at 25 °C in 50 mM sodium phosphate (pH 7.4), 0.1 M KCl, and 2.5 M urea. ∗T119M-TTR was subjected to freeze–thawing cycles to induce dissociation to monomer (see Experimental procedures). The TTR variants tested are indicated in each panel.

TTR variants were incubated at 5 μM and 15 μM in 2.5 M urea for 96 h and subjected to sedimentation velocity analytical ultracentrifugation (AUC) studies to characterize the nature of the denaturation intermediates. The results obtained are summarized in Tables 1 and 2. Under the assayed conditions, sedimentation velocity experiments showed WT-TTR and T119M-TTR at 5 μM behaving as a single species with an experimental sedimentation coefficient of 3.5 S (*s*_20,*w*_= 4.1 S) compatible with the globular tetrameric form of the protein (Fig. 3 and Table 1). When assayed at 15 μM, both WT- and T119M-TTR proteins behave as tetramers, in equilibrium with a small fraction of higher-order soluble oligomers, accounting for 4.3% and 2.5% of total protein, respectively (Table 2). As previously reported, to trigger its dissociation to monomer, T119M-TTR was subjected to freeze–thawing cycles after the 96 h incubation with urea (30). Under these conditions, besides the peak corresponding to the T119M-TTR tetramer (*s*_20,*w*_ = 4.1 S), a second peak was detected at 1.41 S (*s*_20,*w*_ = 1.66 S) compatible with a globular monomer (frictional ratio f/f_0_ = 1.22) (Fig. 3 and Table 1).

**Table 1:** Normalized sedimentation coefficient in water at 20 °C (sw20)

2.5 M urea	c(sw_20_) monomer	c(sw_20_) tetramer
WT-TTR	-	4.12 ± 0.07
L55P-TTR	1.41 ± 0.11	4.08 ± 0.04
R34T-TTR	1.30 ± 0.07	4.06 ± 0.13
K35N-TTR	1.47 ± 0.01	4.07 ± 0.02
K35T-TTR	1.40 ± 0.08	4.18 ± 0.07
R34G-TTR	1.49 ± 0.01	4.07 ± 0.05
T119M-TTR^a^	1.66 ± 0.08	4.10 ± 0.06

Samples were incubated prior measurements for 96 h at 5.0 μM tetramer concentration in 50 mM sodium phosphate (pH 7.4), 0.1 M KCl and 2.5 M urea.

a T119M-TTR was subjected to freeze–thawing cycles to induce tetramer dissociation to monomer.

**Table 2:** Percentage of species determined by analytical ultracentrifugation after 96 h incubation in 50 mM sodium phosphate (pH 7.4), 0.1 M KCl, and 2.5 M urea

2.5 M urea	Monomer		Tetramer		Higher-order oligomers	
	15 μM	5 μM	15 μM	5 μM	15 μM	5 μM
WT-TTR	0.0%	0.0%	95.7%	100.0%	4.3%	0.0%
L55P-TTR	10.4%	16.6%	50.8%	44.4%	38.8%	39.0%
R34T-TTR	3.2%	8.1%	81.9%	79.5%	14.9%	12.4%
K35N-TTR	9.2%	15.9%	63.7%	57.9%	27.1%	26.2%
K35T-TTR	12.2%	20.1%	46.0%	43.7%	41.8%	36.2%
R34G-TTR	21.0%	29.3%	59.5%	59.9%	19.5%	10.8%
V30M-TTR	15.2%	33.7%	59.5%	54.3%	25.3%	12.0%
T119M-TTR	0.0%	0.0%	94.8%	100.0%	2.5%	0.0%

In the case of L55P-TTR and R34-and K35-TTR variants, the sedimentation profile, regardless of the concentration, included a main peak at 3.5 S (*s*_20,*w*_ = 4.1 S) corresponding to the globular tetramer, a second peak at 1.2 S (*s*_20,*w*_ = 1.4 S) compatible with a slightly elongated monomer (f/f_0_ = 1.4), and a variable amount of higher-order oligomers beyond 5 S (Fig. 3 and table 2). Thus, all pathogenic variants exhibited a significant fraction of partially unfolded monomer, absent in the stable WT and T119M forms, and a larger extent of higher-order oligomers than these proteins. These two species are likely interconnected since, for each particular missense mutation, the fraction of monomer decreases as the concentration increases, whereas that of soluble oligomers follows the opposite trend. Most interestingly, intermediate species that populates the urea denaturation reaction between 1.0 and 3.5 M urea displayed ThT-binding ability (Fig. S6). In the 1.0–2.0 M urea range, the R34- and K35-TTR variants exhibited the highest fluorescence emission, equal to or higher than L55P-TTR and much higher than WT-TTR.

### Effect of R34G and K35T mutations in the context of a kinetically stabilized T119M-TTR tetramer

**Figure 4:**
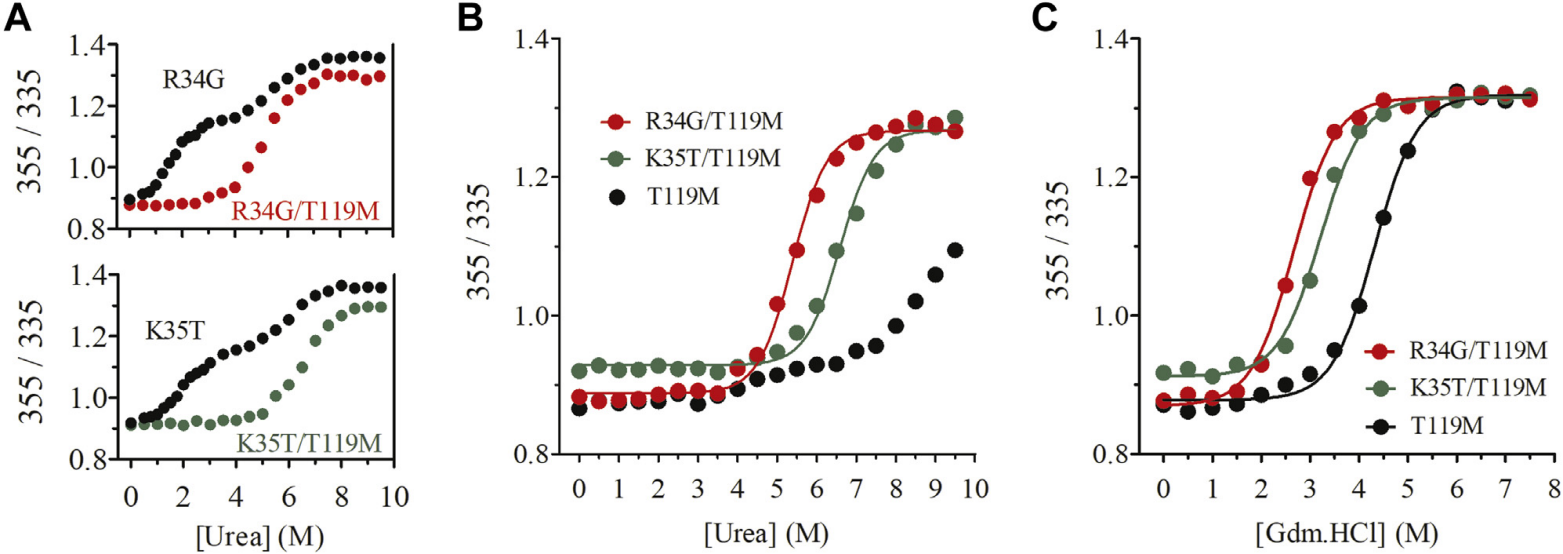
**TTR double mutants chemical equilibrium denaturation**.1.5 μM of T119M-TTR or double mutants R34G/T119M and K35T/T119M TTR were incubated for 96 h at 25 °C in 50 mM sodium phosphate (pH 7.4), 0.1 M KCl with increasing urea or Gdm.HCl concentrations prior measurements of tryptophan fluorescence emission spectra. *A*, comparison of urea equilibrium denaturation curves of R34G with R34G/T119M TTR (*upper panel*) and K35 with K35T/T119 TTR (*lower panel*). *B*, urea denaturation curves of T119M, K35T/T119M and R34G/T119M. *C*, Gdm.HCl denaturation curves of T119M, K35T/T119M, and R34G/T119M.

The TTR R34 and K35 residues are located on the opposite side of the dimer–dimer interface and more distant from the central cavity than L55. Despite this, R34G and K35T variants are as amyloidogenic as L55P at neutral and acidic pH. Therefore, we speculated that these missense variants trigger a long-range destabilization of both populations of TTR tetramers. To test this, the dimer–dimer interface was stabilized with the well-characterized T119M mutation (22, 30, 31) by generating the double mutants R34G/T119M- and K35T/T119M-TTRs. This should allow determining the impact of the missense mutations on a homogeneous population of kinetically stabilized tetramers. The proteins were expressed, purified, and subjected to urea equilibrium denaturation experiments. As is shown in Figure 4*A*, the first urea-induced transition is completely abolished, and the single transition observed starts at a urea concentration similar to that characteristic of the second transition observed in the single mutants (see Fig. 4 for comparison). This indicates that both tetramers and stabilized tetramers dissociate and unfold between 5.0 to 8.0 M urea in these sequence-stabilized variants. Figure 4*B* represents the double mutants plotted in the same graph together with T119M-TTR, which is kinetically stabilized and does not reach equilibrium after 96 h incubation (22). R34G or K35T missense mutations destabilize the TTR tetramer harboring the T119M mutation, promoting a single dissociation/unfolding transition shifted to lower urea concentrations (Fig. 4*B*).

Gdm.HCl-induced equilibrium denaturation revealed that the double mutants exhibited lower stabilities relative to T119M-TTR, all displaying a single transition, with midpoints Cm= 4.3 M for T119M, Cm = 2.6 M for R34G/T119M, and Cm = 3.2 M for K35T/T119M (Figs. 4*C* and S7). Noteworthy, the Gdm.HCl denaturation midpoint of T119M-TTR was lower than WT-TTR (Cm 4.3 vs. 5.1, respectively). In addition, double mutants' transition midpoints were also lower than those of single mutants (see Fig. S7 for comparison).

Overall, K35T and R34G shifted the urea and Gdm.HCl single transition midpoints in the context of T119M (Fig. 4, *B* and *C*), indicating a robust long-range destabilization of the otherwise kinetically stabilized tetrameric interface.

### Molecular dynamic simulations of the TTR variants involving residues 34 and 35

**Figure 5:**
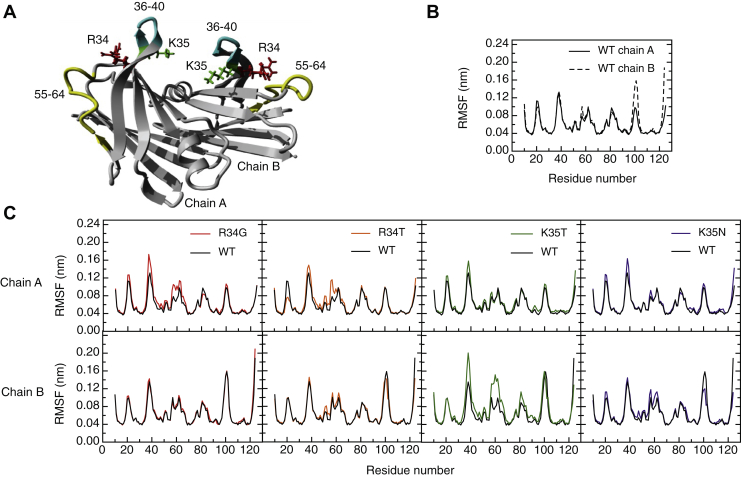
**Effect of TTR mutations on protein dynamics.***A*, location on the 3D structure of the regions with different flexibility patterns. Side chains of residues R34 (*red*), 35 (*green*), and loop regions 36–40 (*cyan*) and 55–64 (*yellow*) are shown. *B*, the per-residue Cα RMSF profiles comparison for the WT-TTR chain A and chain B. *C*, the per-residue Cα RMSF profiles for each pairwise comparison between WT and mutant variants for chain A (*upper panel*) and chain B (*lower panel*) are represented.

To gain insights into the pathogenic mechanism, we studied the impact of mutations at positions 34 and 35 by molecular dynamics (MD) simulations. All-atom explicit solvent MD simulations were carried out to evaluate the changes in the dimer's flexibility on the short 10-ns timescale. The flexibility profiles were computed as per-residue Cα Root Mean Square Fluctuation (RMSF). The RMSF profiles of both WT-TTR monomers (chain A and B) were almost perfectly superimposable with the only exception of the region 96–104 (Fig. 5), which is a solvent-exposed loop not in direct contact with the monomer–monomer interface. The TTR variants structures were generated by virtual mutagenesis and their RMSF profiles compared with the reference WT structure. Regions with higher flexibility were mapped on the 3D structure (Fig. 5). The RMSF profiles of each monomer for WT- and TTR variants were compared individually to appreciate specific differences better. In good agreement with the thermodynamic data, the larger changes in dynamics were observed for the R34G and K35T variants. A local effect, resulting in increased flexibility of the BC loop (residues 36–40), was evident for the four variants. Also, a longer-range effect impacting the flexibility of the DE loop region comprising residues 55–64 was observed; this might explain why the impact of some of these mutations resembles that of L55P in terms of aggregation and amyloid propensity. Surprisingly, R34G impacted mostly the first monomer's dynamic (chain A), whereas K35T essentially affected the second one (chain B). To confirm this differential impact of the mutations on the TTR protomers, the frequency of side chain residue–residue contacts was evaluated along the simulation (see experimental procedures). In agreement with the RMSF profiles, the contact analysis for the BC loop region revealed that R34G establishes less persistent contacts in subunit A, whereas K35T shows a marked decrease in the frequency of contacts in subunit B (Fig. S8). Similar findings were observed for the DE loop region, with K35T causing a marked reduction in the frequency of contacts in subunit B, while R34G diminishes the contact frequencies of subunit A (Fig. S9). The molecular reasons behind these different behaviors are not evident, but what it comes evident is that, according to the MD simulations, the impact of the mutations is restricted to the BC and DE loops surroundings.

### Structural basis for TTR destabilization induced by variants at residues 34 and 35

The crystal structures of a significant number of TTR pathogenic mutants, including L55P, have been solved (32, 33). However, at neutral pH, they are all virtually identical to that of WT-TTR. To decipher the structural determinants accounting for the high amyloidogenicity of the R34G and K35T TTR variants, we decided to explore the conformation of the region surrounding the mutation sites under destabilizing conditions using X-ray diffraction. R34G and K35T TTR mutants readily aggregate at mildly acidic pH, and therefore their structural properties cannot be accessed in these proamyloidogenic conditions. We took profit of the fact that R34G/T119M- and K35T/T119M-TTR double mutants do not aggregate significantly at acidic pH (Fig. S10) to solve their crystal structures at pH 5.5 at 1.5 and 1.4 Å resolutions, respectively. We also solved the crystal structure of the nonamyloidogenic variant T119M-TTR at 1.3 Å resolution at the same pH as a control.

**Figure 6:**
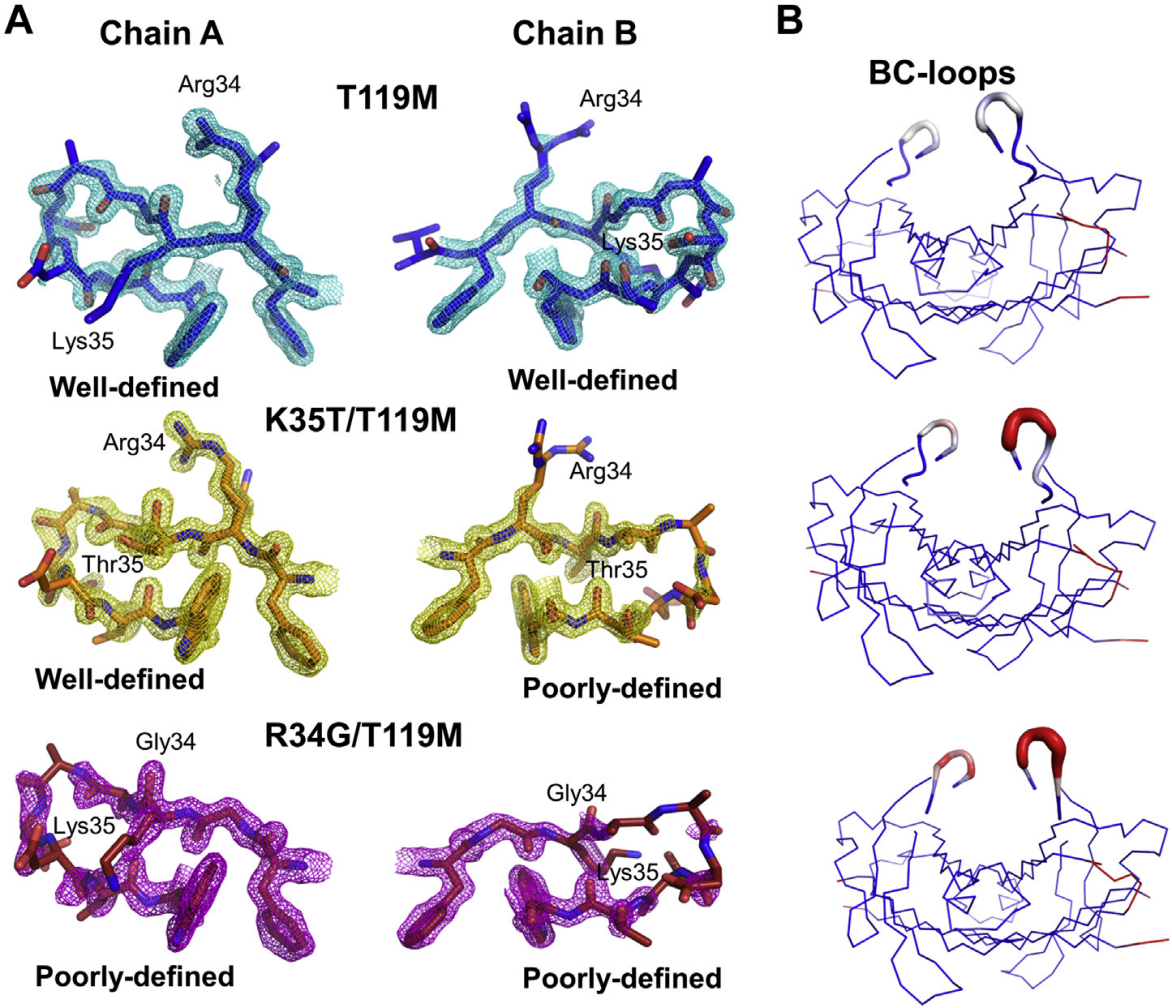
**Structural features of T119M, R34G/T119M, and K35T/T119M TTRs.***A*, stick representation showing the BC-loop regions of TTR dimers with electro densities maps countered at 1.5σ. *B*, Putty cartoon of B-factor variation on the A/B dimers, colored from low to high (*blue* to *red*). Side chains of mutated residues are depicted as *sticks*. Rendered using PyMOL.

As expected, no major structural differences between the pathogenic mutants and T119M-TTR were observed, with overall Root Mean Square Deviation (RMSD) values for the Cα of 0.24 Å for R34G/T119M-TTR and 0.15 Å for K35T/T119M-TTR (Fig. S11). The crystallographic parameters for both variants are shown in Table S1. As observed for other TTR crystals, the complete polypeptide chains could be traced, except for the terminal residues, 1–9 and 126–127, disordered and not defined in the electron density. We also could not trace the residues 101–102 from chain B in all three structures. The well-reported aggregation-prone CD-, DE-, EF-, and FG-loop regions (residues: 49–53, 56–66, 74–90, and 98–104, respectively) were not affected by R34G and K35T mutations. On the other hand, in good agreement with the molecular simulations, the BC-loop regions (residues 36–40) were remarkably affected in the double mutants, in one or both asymmetrical chains. Indeed, putty-style cartoon representations based on mean Cα β-factors show much greater values in these regions, pointing them as the most dynamic in K35T/T119M and R34G/T119M TTR (Figs. 6 and S12). It is worth mentioning that the higher flexibility of the BC loop in both chains of R34G matches with its higher instability revealed by the thermodynamic analysis. The pattern and the distances of main-chain H-bonds that pack the B strand to C and E strands were well conserved between the double mutants and T119M TTR, except for the one established by Lys35 and Ile68, which become more sparse: T119M (2.81, 2.86), K35T/T119M (2.87, 3.07) to R34G/T119M (2.93, 3.26). The contact region at the tetrameric interface of double mutants, which consists of hydrophobic interactions between the AB- (residues 18–28) and the GH loop (residues 105–122), was preserved. The presence of Met119 keeps all those interaction residues virtually at the same positions, which allow the formation of key contacts in the presence of the second amyloidogenic mutation. The dimerization interfaces were also preserved on all crystal structures.

Overall, the crystallographic data suggest that BC-loop fluctuations constitute the triggering mechanism for R34G and R35T tetramer dissociation and subsequent aggregation.

## Discussion

Pathogenic mechanisms of distinct disease-associated mutations of TTR have been extensively studied for more than 25 years (22, 34, 35, 36). It is well accepted that they affect the tetramer affinity, tetramer dissociation rates (rate-determining step for amyloid fibril formation), and/or the thermodynamics of misfolding of monomeric TTR (21). Nevertheless, it is still unclear why a given mutation is associated with TTR deposition in a specific tissue or organ and connected to a particular clinical manifestation. This is best illustrated by the R34G, R34T, K35N, and K35T TTR familial mutations we study here. R34G- and K35T-TTR variants cause vitreous amyloidosis (24, 25), whereas R34T and K35N mutations result in amyloid polyneuropathy and restrictive cardiomyopathy (26, 27). Therefore, in both cases, a mutation in the same position results in two different clinical phenotypes. Besides, the mutation of a positive residue to the same amino acid (R34T, K35T) also gives rise to different disease manifestations, even if these positions are adjacent in the sequence. We address the conformational origin of these differences here.

Several *in vitro* approaches have been used to evaluate the effect of single-point mutations on TTR conformational stability. One of them is acid-mediated denaturation, as lowering pH induces tetramer dissociation and triggers the conformational arrangement needed for fibril formation (21). The optimal pH to form amyloid fibrils *in-vitro* for WT-TTR is pH 4.4, while disease-causing variants usually dissociate and form fibrils at higher pHs and/or to a much greater extent. The R34- and K35- variants studied here are more sensitive than WT-TTR to pH-induced dissociation. Remarkably, the pH range over which dissociation and amyloid formation occurred was close to physiological pH, particularly in the case of the eye deposited R34G- and K35T-TTR variants, more aggregation-prone than the R34T- K35N-TTR polyneuropathic/cardiac variants in all assayed conditions. These findings suggest that R34G- and K35T-TTR could dissociate and form amyloid fibrils near physiological conditions. Indeed, these two mutants' behavior resembles the one of L55P-TTR (28), the most pathogenic mutant described so far. The high susceptibility to aggregation promoted by these mutations is surprising since these TTR residues are among the more distant from the dimer–dimer interface, where the initial tetramer dissociation necessarily starts.

The thermodynamic stability of disease-associated TTR variants has been often assessed by equilibrium urea denaturation curves, comparing a single transition midpoint of dissociation/unfolding relative to the WT-TTR (C_m_ = 3.2 at 1.5 μM) (35). In most cases, they are thermodynamically destabilized, displaying a Cm < 3.2 (29, 33). In the case of WT-TTR, tetramer dissociation and monomer unfolding have been proposed to be thermodynamically linked. Supporting evidence for this mechanism comes from studying an engineered nonamyloidogenic monomeric variant of TTR (M-TTR: F87M:L110M), harboring identical secondary and tertiary structures to that of tetrameric TTR at pH 7.0 (36). Urea and Gdm.HCl denaturation curves of M-TTR and tetrameric TTR followed by tryptophan fluorescence showed nearly identical transitions, beginning to dissociate-unfold above 2.5 M urea, and considered to be fully unfolded beyond 4.0 to 5.0 M urea. In our case, a detailed inspection of the conformational stability of WT-TTR as measured by urea denaturation revealed that a second transition starts above 8.0 M urea, without reaching a stable unfolded baseline even beyond 9.0 M urea after 96 h incubation. Several factors have been reported to increase the kinetic barrier of dissociation, making tetramer dissociation slower. Two Lys residues (Lys 15 and Lys 15′) from neighboring subunits, projecting into the dimer–dimer interface, repel each other destabilizing the tetramer. The binding of negatively charged ions to these positively charged Lys residues drastically increases tetramer stability (29). It was shown that increasing concentrations of KCl stabilizes the tetramer, rendering biphasic denaturation curves similar to the one we obtained for WT-TTR (29). Our last purification step consisted of a size-exclusion chromatography containing 0.1 M KCl, and the urea equilibrium denaturation was performed in the presence of 0.1 M KCl. Because we consistently obtained biphasic WT-TTR denaturation curves, one possible explanation is that a subpopulation of TTR is loaded with an anion (Cl^−^) along the purification process. However, based on the present data, we cannot rule out another mechanism that leads to the stabilization of tetrameric TTR.

**Figure 7:**
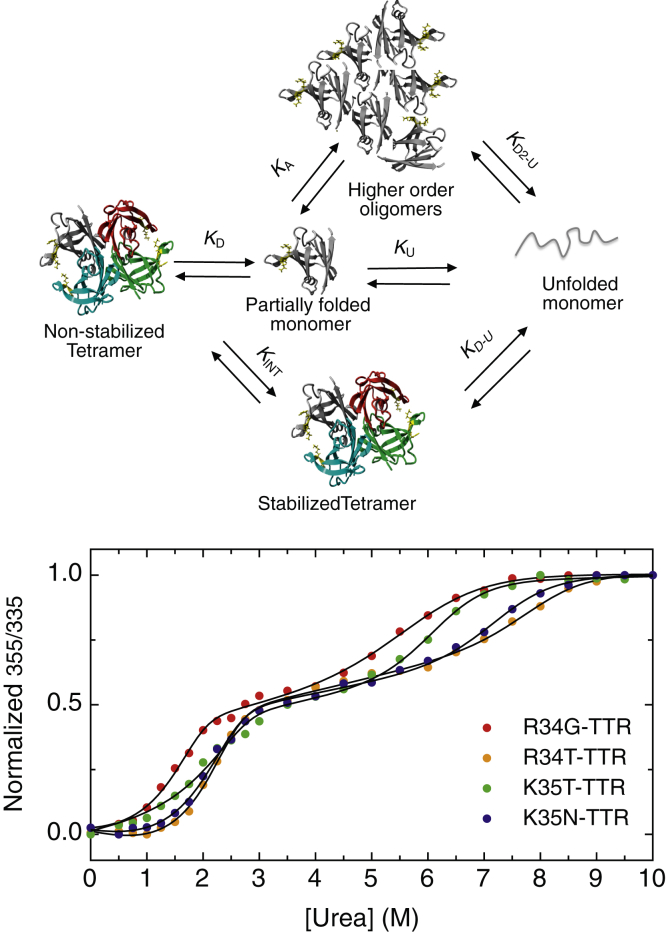
**Model for urea denaturation mechanism of TTR variants at residues R34 and K35.** Urea denaturation curves followed by tryptophan fluorescence of TTR variants display two transitions. The first one takes place between 1.0 and 3.0 M urea and corresponds mainly to nonstabilized tetramer dissociation (*K*_*D*_) to partially folded monomers. This misfolded monomeric species can either self-assemble (*K*_*A*_) into higher-order oligomers or unfold (*K*_*U*_) at increasing urea concentrations. The second apparent transition occurs between 5.0 and 8.0 M urea and likely represents a population of kinetically stabilized tetramers that do not reach equilibrium on the experimental timescale. Higher-order oligomer disassembly and monomer unfolding could also occur within this urea range (*K*_*D2-U*_). Interconversion between nonstabilized and stabilized tetramers (*K*_*INT*_) by subunits exchange would be very slow due to the high kinetic barrier of dissociation of stabilized tetramer.

The single-point mutations studied in the present work uncovered a complex urea denaturation pathway, showing two transitions that were affected differently depending on the variant (Fig. 7). As mentioned above, we assume that two different populations of TTR tetramers coexist; nonstabilized tetramers and stabilized tetramers. The first equilibrium transition represents nonstabilized tetramer dissociation, monomer partial unfolding, and monomer unfolding, whereas the second transition would correspond to stabilized tetramers that dissociate very slow in urea and do not reach equilibrium within 96 h incubation. Hence the second transition would be an apparent transition and not an equilibrium transition. Physical evidence by AUC experiments revealed that in 2.5 M urea, the main species populating the first posttransition region are tetramers and extended monomers. Interestingly, higher-order oligomers were also detected, raising the possibility that partially folded monomers can self-assemble into oligomers, even in the presence of urea. It is very likely that the low concentration of urea at which tetramer dissociation occurs is insufficient to trigger monomer unfolding and allows the accumulation of misfolded monomers that arrange into oligomers. Additionally, we found that in the urea concentration range at which the first transition occurs, the mutant proteins bind Th-T, indicating the presence of intermediates with amyloid-like properties, which likely correspond to the observed high-order oligomers.

In agreement with our results, the TTR acid denaturation pathways of WT-, V30M-, and L55P-TTR variants were previously characterized by AUC (37). A monomer with an alternatively folded tertiary structure was identified, which self-assembles into a ladder of quaternary structural intermediates to form amyloid fibrils (37). In addition, resveratrol-binding experiments support the coexistence of two populations of tetramers, with different stabilities. In concordance with our findings, a similar biphasic urea curve was described for V30M TTR, with denaturation intermediates populating the first posttransition region able to bind resveratrol (35). Interestingly, after incubation of V30M TTR for 2 weeks in urea, Hurshman Babbes *et al*. showed that the amplitude of the first transition increases, whereas the amplitude of the second transition decreases, compatible with our proposed model of two populations of tetramers coexisting (35). When chemical denaturation was performed using Gdm.HCl, only one transition is observed, corresponding to a linked dissociation-unfolding process, as previously reported (38). In this case, chloride ions saturate their binding sites, resulting in a homogeneous population of highly stable tetramers populating the pretransition region (29).

In urea denaturation experiments, R34- and K35- mutations significantly decrease the C_m_ for both the first and the second transitions, with R34G- and K35T-TTR exerting the most substantial destabilizing effect, as confirmed by the C_m_ of the single transitions observed in Gdm.HCl denaturation experiments, and in excellent agreement with the relative proaggregational effect of the different mutations at mildly acidic pH. Strikingly, the R34G-TTR first transition occurs at a lower urea concentration than the single broad transition of L55P-TTR, indicating that this vitreous amyloidosis-associated mutant is highly destabilized. Indeed, AUC experiments indicate that R34-and K35- variants and L55P-TTR all dissociate significantly to a misfolded aggregation-prone monomer under conditions where WT-TTR still exhibits a stable tetrameric structure.

Despite the R34 and K35 residues at the C-terminus of TTR β-strand B are distant from the dimer–dimer interface, the data indicated that their mutation should impact the stability of the intermolecular contacts in this TTR tetramer region. To confirm this long-range destabilizing effect, we exploited the T119M mutation. T119M (22, 30), R104H (39), and A108V (40) are among the best characterized natural TTR tetramer stabilizing mutations, all acting by increasing the kinetic barrier of dissociation (41). When the T119M mutation was introduced together with either R34G or K35T, the first transition at low urea concentration was completely abolished. We assume that a homogeneous population of sequence-induced stabilized tetramers populate the pretransition region. However, even in the highly stabilized T119M context, the introduction of the R34G and K35T mutations strongly destabilizes the tetramer, shifting the midpoint transitions to lower urea or Gdm.HCl concentrations, again with a higher impact of the R34G substitution, confirming in both cases a long-range destabilizing impact that facilitates tetramer dissociation. It is worth mentioning that the urea range at which anion-stabilized tetramers and T119M stabilized tetramers dissociate fairly coincides (Fig. 4*A*). By using distinct approaches, several reports agreed that T119M-TTR tetramers and monomers have thermodynamic stabilities similar to their WT-TTR counterparts (30, 42). Counterintuitively, by Gdm.HCl denaturation experiments, we found that tetramers harboring Met at position 119 were less stable than those with Thr (see Fig. S7). One plausible explanation for this behavior is that the specific residue at position 119 may differently affect tetramer stabilization mediated by Cl^−^, although this effect should be further confirmed. In our hands and in agreement with previous reports, Met at position 119 protects TTR from acid and urea-induced denaturation, by increasing the kinetic barrier of tetramer dissociation in these conditions (Figs. 4*B* and S10).

The kinetically stabilized double mutants were crystallized at pH 5.5, allowing to rationalize the structural basis for pH-induced tetramer destabilization. Although the crystallographic structures of the double mutant variants were very similar to that of T119M-TTR at the same pH, greater flexibility of the BC-loop (higher Cα β-factors) was observed in the presence of the K35T and, especially, the R34G mutations. This region becomes the most dynamic in the double mutant structures, in excellent agreement with the individual mutants studied by MDs simulations, suggesting that transient local unfolding events at the BC strands are likely. In addition to the crystallographic and MD evidences of a higher BC loop dynamism triggered by R34 and K35 mutations, the simulations also suggest that the variants may indirectly impact the DE loop region flexibility, including L55, which might explain why the most aggressive of these mutants resemble L55P-TTR regarding their aggregation and amyloid propensity *in vitro*. This dynamic signature of the DE loop was not observable in static-X ray structure, which might respond to the drastically different environment that the system experiences in crystallographic experiments with respect to MD simulations, where the system is in solution and not conformationally constrained in crystal form. Notably, the relative destabilizing effect of the different 34 and 35 variants cannot be read directly from the static TTR structure at physiological pH without considering dynamics since structure-based computational tools wrongly classified K34T-TTR to be the most destabilized of the mutants (43).

Overall, our data indicate that the specific residue at position 34 or 35 determines the BC-loop flexibility. An increase in this TTR region dynamism triggers long-range destabilization of the dimer–dimer interface, which in turn promotes tetramer dissociation and the accumulation of an aggregation-prone misfolded monomer that assembles into oligomers. Other TTR variants were shown to destabilize the native tetrameric structure by enhancing loop flexibility. For instance, single-point mutations G53A and S52P reduce the thermodynamic stability by perturbing the CD loop (44).

The degree of BC-loop dynamism induced by TTR mutations at positions 34 and 35 is associated with their destabilizing impact and the aggregation propensity of the variant. According to our data, for the studied TTR variants, vitreous amyloidosis occurs when the combination of the mutated position and the identity of the substituent residue results in a high dynamism and destabilization, which endorses the variant with increased amyloidogenicity.

Importantly, the introduction of the T119M mutation in the context of R34 and R35-TTR variants prevents aggregation. Thus, it is expected that kinetics stabilizers that mimic the T119M stabilizing effect, such as Tafamidis (17, 45), Diflunisal (46), Tolcapone (20, 47), or AG10 (48, 49), would become effective drugs to treat the TTR amyloidosis associated with BC-loop fluctuations.

## Experimental procedures

### Protein expression and purification

WT-TTR was cloned into pET28A vector without any fusion tag. R34G-; R34T-; K35N-; K35T-; T119M-; R34G/T119M-; and K35T/T119M- TTR variants were prepared by standard site-directed mutagenesis protocols using WT-TTR pET28A as template. TTR mutations were confirmed by Sanger sequencing. L55P and V30M TTR expression vectors were kindly provided by Prof. Debora Foguel. All TTR variants were recombinantly expressed in BL21(DE3) as soluble proteins and purified from cell supernatants following previously described procedures (20). Briefly, soluble fractions were treated by two consecutive steps of ammonium sulfate precipitation (50% and 90%, respectively). The 90% precipitate was solubilized in 25 mM Tris.HCl (pH 8.0) and extensively dialyzed against the same buffer. Samples were loaded onto a Hi-Trap Q HP column (GE Healthcare, Chicago, IL, USA) equilibrated in 25 mM Tris HCl (pH 8.0) and eluted with a ten CV linear gradient from 0 to 0.5 M NaCl. The TTR-enriched fractions were precipitated in 90% ammonium sulfate, and the precipitate was dissolved in 10 ml of 25 mM Tris.HCl (pH 8.0), 100 mM KCl. TTR enriched samples were finally purified by gel filtration chromatography onto a HiLoad 26/600 Superdex 75 prep-grade column (GE Healthcare) equilibrated in 20 mM Tris.HCl (pH 8.0), 100 mM KCl. The concentrations of all protein solutions were determined spectrophotometrically at 280 nm, using a molar extinction coefficient of λ_280 nm =_ 77.600 M^−1^ cm^−1^ for the TTR tetramer.

### Aggregation and fibril formation assays

Two approaches were used to evaluate amyloid fibril formation: (1) Turbidity assay, which measures the presence of large insoluble aggregates. (2) Thioflavin T (ThT)-binding assay that measures the presence of either amyloid fibrils or soluble amyloid-like small oligomers. TTR variants (3.5 μM) were incubated under quiescent conditions in a broad range buffer (100 mM Tris.HCl, 50 mM MES, 50 mM sodium acetate, 0.1 M KCl) ranging from pHs 4.0 to 7.0 for 72 h at 37 °C. The ionic strength of the buffer is kept constant throughout the pH range tested (48). After the incubation the samples were vortexed and optical density was measured at 330 nm on a UVI-Vis Carry spectrophotometer (Shimadzu). Fibril formation was also assessed by ThT binding incubating 1 μM of the sample (vortexed to achieve homogeneity) with 25 μM ThT in 100 mM Tris·HCl (pH 8.0), 0.1 M KCl. The mixed sample was then excited at 440 nm and emission at 482 nm was recorded on a Jasco 8200 spectrofluorometer.

### Transmission electron microscopy (TEM)

Aggregated suspensions were absorbed onto 200-mesh carbon-coated copper grids for 5 min and then blotted to remove excess material. Negative staining was performed by adding 5 μl of 2% (w/v) uranyl acetate. Samples were dried on air for 3 min. The grids were imaged with a Jeol 1200 electron microscope (Jeol Ltd) operating at a60 kV acceleration voltage.

### Chemical equilibrium denaturation of TTR variants

Urea and guanidinium chloride (Gdm.HCl) denaturation curves were performed incubating 1.5 μM of TTR tetramer in 50 mM sodium phosphate (pH 7.4), 0.1 M KCl with varying concentrations of chaotropes. Samples were incubated at room temperature for 96 h before fluorescence measurements.

Tryptophan fluorescence was used to monitor TTR tertiary structural changes as a function of denaturant concentration. The samples (25 °C) were excited at 295 nm, and three accumulations of the fluorescence emission spectra were taken from 310 to 410 nm on a Jasco 8200 spectrofluorometer. Fluorescence emission data were analyzed by first subtracting the buffer background at the appropriate denaturant concentration, and the center of spectral mass of the emission spectrum was quantified as follows:

(1) $$\text{CM}\;\left({\text{cm}}^{-1}\right)=\sum \left(\text{υi.Fi}\right)/\sum \text{F}\;\text{i}$$

where F i is the fluorescence emission at wave number υi, and the summation is carried out over the range of measured values of F.

The binding of resveratrol to the TTR tetramers was performed to quantitatively assess their quaternary structure stabilities as a function of urea concentration, as previously reported (22). After measuring the fluorescence emission spectra, the samples at 1 μM were mixed with 18 μM resveratrol and fluorescence emission spectra were obtained with excitation and emission wavelengths of 320 and 394 nm, respectively. To quantify the concentration of TTR tetramer as a function of urea concentration, resveratrol-binding curves were performed for each TTR variant (0–2.0 μM TTR tetramer) by using 18 μM of resveratrol (see Fig. S4). The fluorescence intensity at 394 nm was plotted versus the concentration of TTR, exhibiting a linear fit as was previously reported (22).

### Sedimentation velocity assays (SV)

TTR samples at 5 and 15 μM were incubated during 96 h in 50 mM sodium phosphate pH 7.0, 100 mM KCl, and 2.5 M urea. After incubation, samples were centrifuged at 12.100*g* for 15 min to remove insoluble aggregates. The remaining soluble protein was quantified spectrophotometrically at 280 nm and the percentage of proteins that remain in solution after centrifugation ranged from 90 to 100%. TTR samples were then loaded (400 μl) into analytical ultracentrifugation cells. The experiments were carried out at 25 °C and 48,000 rpm in a XL-A analytical ultracentrifuge (Beckman-Coulter Inc) equipped with an UV-VIS absorbance detection system, using an An-50Ti rotor, and 12 mm Epon-charcoal standard double-sector centerpieces. Sedimentation profiles were recorded at 280 nm. Differential sedimentation coefficient distributions were calculated by least-squares boundary modeling of sedimentation velocity data using the continuous distribution c(*s*) Lamm equation model as implemented by SEDFIT (50). These *s* values were corrected to standard conditions (water, 20 °C, and infinite dilution) (51) using the program SEDNTERP (52) to get the corresponding standard *s* values (*s*_20,*w*_).

### Crystallography and structure determination

Crystals of R34G/T119M, K35T/T119M and T119M TTR variants were obtained at 18 °C by hanging-drop vapor diffusion methods after purification and concentration. The reservoir solution contained between 7% glycerol, 1.3 M sodium citrate, pH 5.5. Single crystals appeared after 3 days from equal volumes of protein solution (0.1 mM in 50 mM Tris·HCl pH 8.0, 100 mM KCl, and 1 mM EDTA) and reservoir solution. Crystals were cryo-protected in reservoir buffer containing 12% glycerol and directly flash-frozen in liquid nitrogen prior to diffraction analysis. Diffraction data were recorded from cryo-cooled crystals (100 K) at the BL13-XALOC beamline from ALBA synchrotron (53). Data were integrated and merged using XDS (54) and scaled, reduced, and further analyzed using CCP4 (55) (Table S1). The structures of TTR variants were determined from the X-ray data by molecular replacement using a former TTR structure (PDB 1F41) as a model using the program Phaser (56). Model refinement and rebuilding were performed with (57, 58). Refinement and data statistics are provided in Table S1, structural representations were rendered using Pymol Software Package. The structures have been deposited with PDB codes 6FWD, 6FZL, and 6FXU.

### Molecular dynamics (MD) simulations

The X-ray structure of wild-type TTR (PDB 1F41) has been used as starting structure for 150-ns explicit solvent MD simulations using the CHARMM22∗ force field (59) and GROMACS software. We simulated the dimeric assembly of the protein. The starting structures for TTR variants (R34G, R34T, K35N, and K35T) have been achieved upon *in silico* mutagenesis with Pymol. We mutated *in silico* the wild-type structure to be able to assess the perturbation induced upon the mutations on the native structure dynamics. Periodic boundary conditions were employed for the simulations and the initial structures were embedded in a dodecahedral box of TIP3P water molecules so that all the protein atoms were at a distance equal or greater than 15 Å from the box edges. To neutralize the overall charge of the system, a number of water molecules equal to the protein net charge were replaced by counterions and a concentration of 150 mM NaCl has been used. The preparation steps have been carried out as previously described (60) and productive MD simulations were performed in the NVT ensemble at 300 K and 1 bar using an external Berendsen bath with thermal and pressure coupling of 0.1 and 1 ps, respectively. The LINCS algorithm was used to constrain heavy-atom bonds, allowing for a 2-fs time step. Long-range electrostatic interactions were calculated using the Particle-Mesh-Ewald (PME) summation scheme. van der Waals and short-range Coulomb interactions were truncated at 10 Å. The nonbonded pair list was updated every ten steps and conformations were stored every 4 ps.

The main-chain RMSD was computed using the initial structure for MD simulations as a reference to assess the stability of the productive simulations. To evaluate the effects induced by the mutations on the monomer–monomer interface, we calculated an average per-residue C-α RMSF profile using time windows of 10 ns for the averaging. We then compared pairwise the RMSF profiles of the WT and mutant TTR variants and mapped the regions with enhanced flexibility upon mutation on the 3D structure. The contact analysis was carried out using a custom Python script, and two residues were considered in contact if they had at least a pair of side chain atoms lying within 4.5 Åfrom each other and a sequence distance greater than 1 (*i.e.*, they were separated by at least one residue along the sequence). The persistence of each contact was calculated by dividing the number of frames of the simulation where the contact was present by the total number of frames. The MD data are available in the OSF (https://osf.io/az7vu/) and GitHub repositories (https://github.com/ELELAB/TTR_gatekeeper) associated with this publication.

## Data availability

The crystal structures have been deposited in Protein Data Bank (https://www.rcsb.org/) with PDB codes 6FWD, 6FZL, and 6FXU. The MD data are available in the OSF (https://osf.io/az7vu/) and GitHub repositories (https://github.com/ELELAB/TTR_gatekeeper) associated with this publication. Additional data that support the findings of this study are contained within the manuscript and supporting information. Any additional information or data are available upon request. Contact information: e-mail: salvador.ventura@uab.es.

## Supporting information

This article contains supporting information.

## Conflict of interest

S. A. E. is a member of the scientific and technological research career of CONICET. The authors declare that they have no conflicts of interest with the contents of this article.
