# Supplementary material for: Disease-associated mutations impacting BC-loop flexibility trigger long-range transthyretin tetramer destabilization and aggregation
Source: J Biol Chem. 2021 Jul 31;297(3):101039. doi: 10.1016/j.jbc.2021.101039 (PMC8406001; doi:10.1016/j.jbc.2021.101039)
Supplement: Table S1 and Figures S1–S12 [file mmc1.pdf]

Supplementary information

Disease-associated mutations impacting BC-loop flexibility trigger long range transthyretin tetramer destabilization and aggregation

Sebastián A. Esperante*[1,], Nathalia Varejão[1], Francisca Pinheiro[1], Ricardo Sant'Anna[1], Juan Román Luque-Ortega[2], Carlos Alfonso[3], Valentina Sora[4,5], Elena Papaleo[4,5], Germán Rivas[3], David Reverter[1]& Salvador Ventura*[1]

Table S1. *Data collection and refinement statistics. Values in parentheses are for the last shell.*

	R34G:T119M TTR	K35T:T119M TTR	T119M TTR
**Data collection**			
Space group	$P2_122_1$	$P2_122_1$	$P2_12_12$
Unit cell parameters (Å) a, b, c	43.13, 63.96, 85.98	43.06, 63.58, 85.96	86.07, 43.18, 63.83
Wavelength (nm)	0.97949	0.97949	0.97949
Resolution range (Å)	63.9-1.58	63.5-1.4	63.8-1.3
Rmerge	0.04(1.24)	0.02(0.77)	0.02(0.88)
Rpim	0.03 (0.74)	0.01(0.50)	0.01(0.56)
$(I/\sigma(I))$	36.6(2.7)	54.4(2.1)	65.3(2.0)
Completeness (%)	100.0(98.3)	100.0(96.6)	100.0(95.4)
Multiplicity	6.2(7.7)	5.7(6.2)	6.7(6.6)
CC (1/2)	0.99(0.68)	0.99(0.73)	0.99(0.59)
**Structure refinement**			
Resolution range (Å)	51.3-1.5	42.9-1.4	63.8-1.3
No. of unique reflections	31,271	41,465	46,691
Rwork/Rfree (%)	0.19/0.22	0.18/0.19	0.19/0.21
No. of atoms			
Protein	1,781	1,805	1,830
Water molecules	214	190	181
Overall B factors ($Å^2$)	23.2	24.5	22.8
R.m.s. deviations			
Bonds (Å)	0.016	0.007	0.007
Angles (°)	1.68	1.04	1.08
**PDB code**	6FWD	6FZL	6FXU

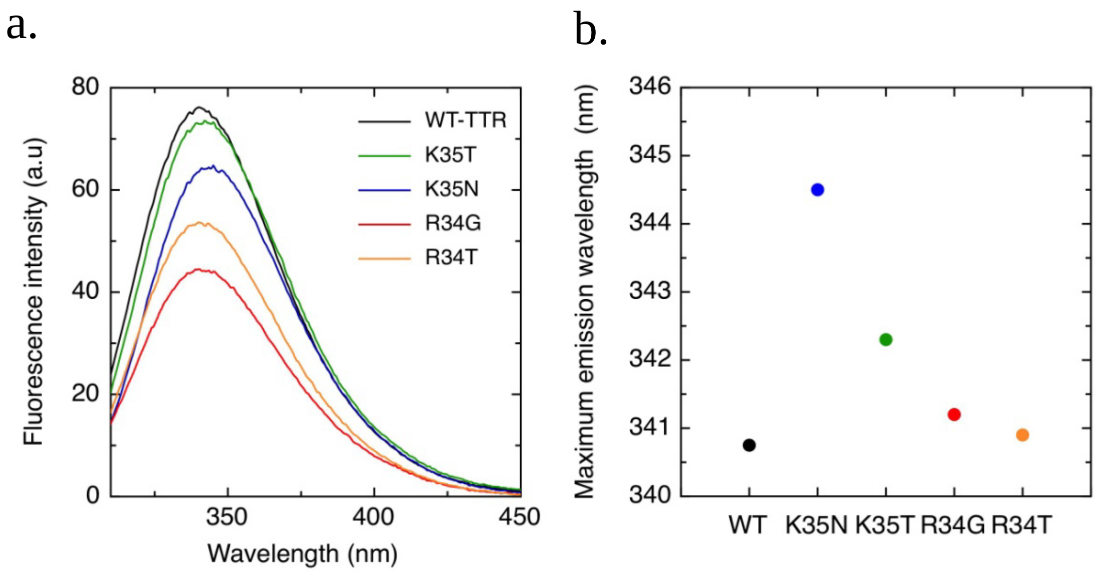

Figure S1. *Tryptophan fluorescence emission spectra of TTR disease associated variants under native conditions.* (a) Tryptophan fluorescence emission spectra of TTR variants at 1.5 µM in 50 mM sodium phosphate (pH 7.4) and 0.1 M KCl. Each TTR variant color code is indicated inside the graph. (b) Maximum emission wavelength of TTR variants under native conditions.

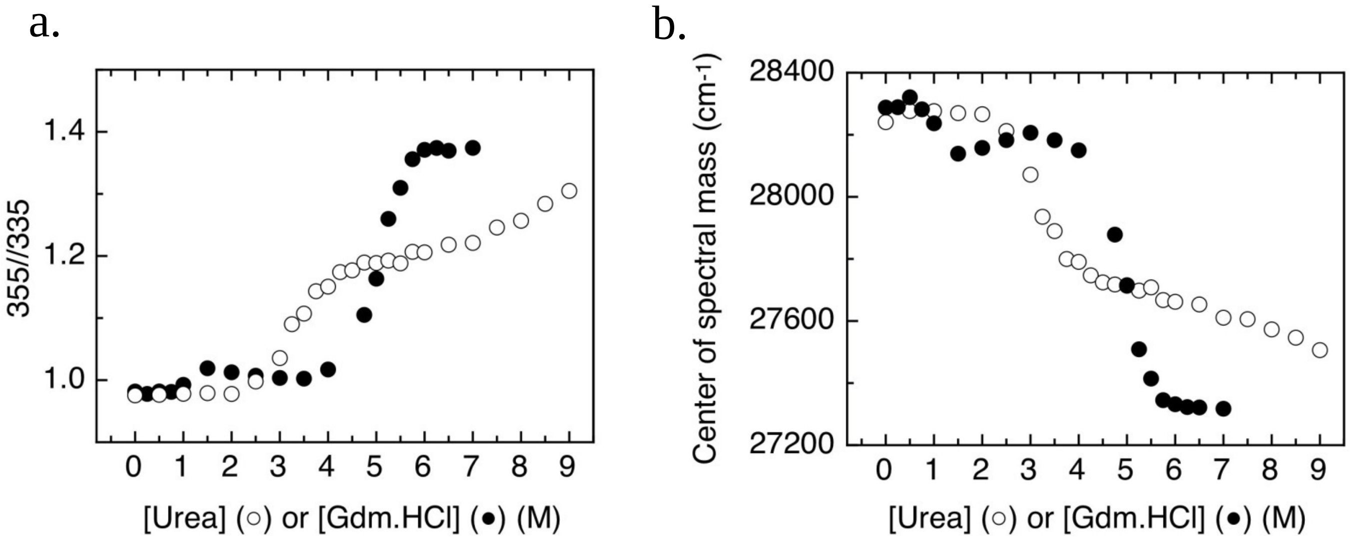

Figure S2. *Guanidinium chloride and urea denaturation of WT-TTR*
1.5 μM of WT-TTR was incubated for 96 h at 25 °C in 50 mM sodium phosphate (pH 7.0), 0.1 M KCl with increasing concentrations of urea (open circles) or Gdm.HCl (filled circles). Tryptophan fluorescence emission spectra were measured as a probe for tertiary structure changes and represented as the 355/335 ratio (a) or the center of spectral mass of tryptophan fluorescence emission spectrum (b).

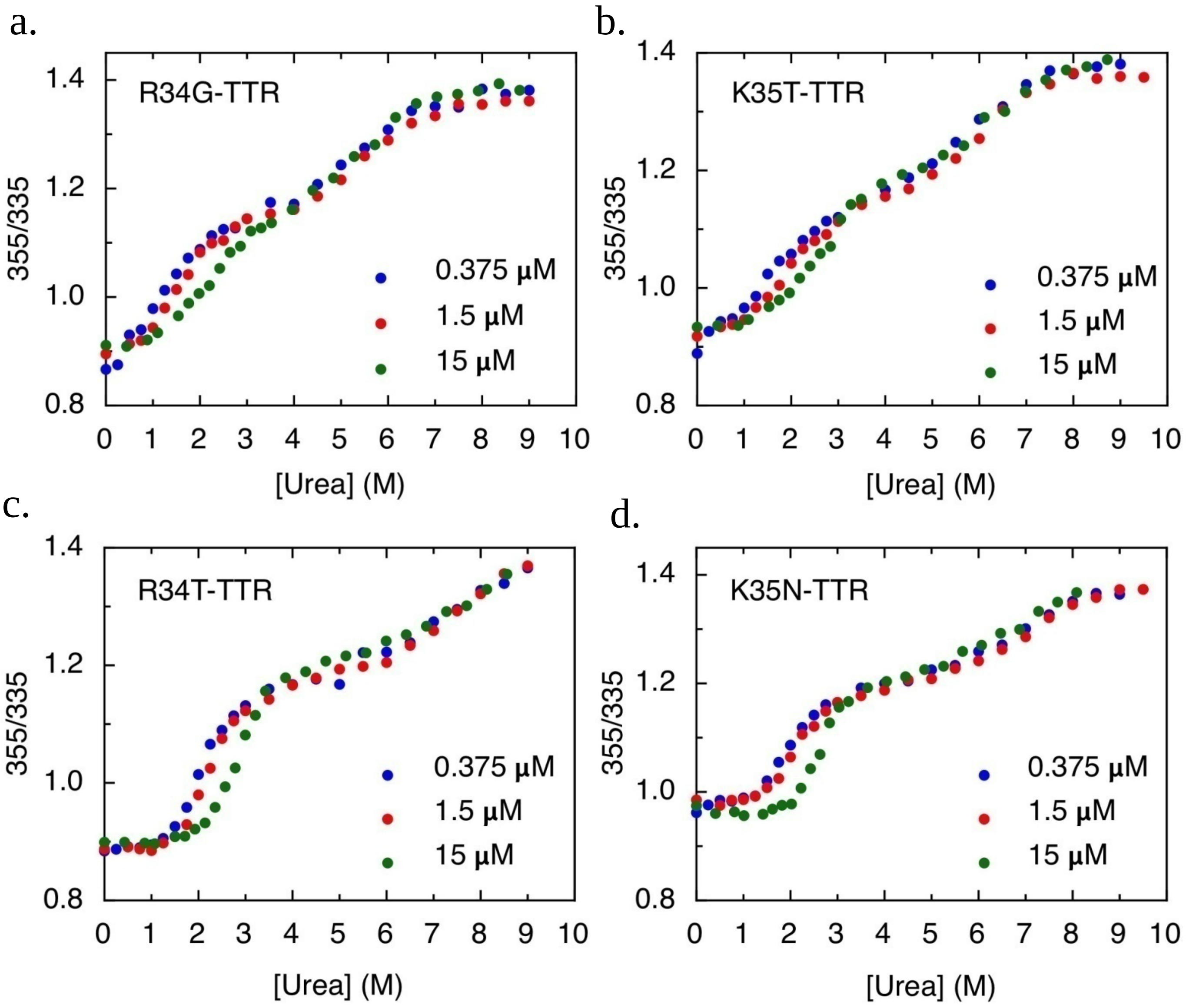

Figure S3. *Concentration dependence denaturation of TTR disease associated variants.*
0.375 µM (blue dots), 1.5 µM (red dots) or 15µM (green dots) of TTR variants were incubated for 96 h at 25 °C in 50 mM sodium phosphate (pH 7.4) containing 0.1 M KCl with increasing urea concentrations prior measurements of tryptophan fluorescence emission spectra. The tryptophan fluorescence emission intensity ratio displayed is defined as the ratio of the tryptophan emission intensity at 355 nm (unfolded state) to the tryptophan emission intensity at 335 nm (folded state) and is used as a measure of foldedness. Panel a) R34G-TTR b) K35T-TTR c) R34T-TTR and d) K35N-TTR,

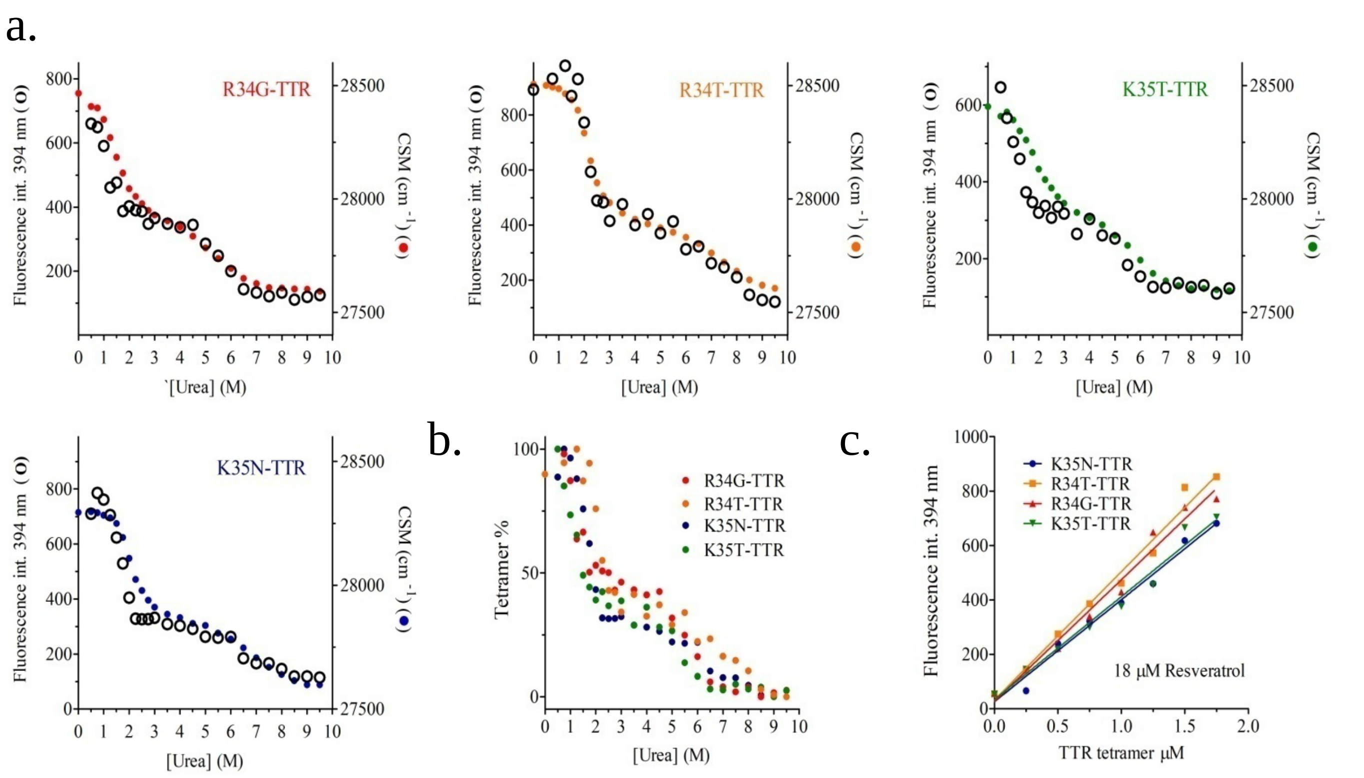

Figure S4. *Urea denaturation coupled tertiary and quaternary structure changes.*
1.5 μM of TTR variants were incubated for 96 h at 25 °C in 50 mM sodium phosphate (pH 7.4), 0.1 M KCl with increasing urea concentrations prior measurements. Tryptophan fluorescence emission spectra were measured as a probe for tertiary structure changes. After tryptophan fluorescence measurements, 18 μM reveratrol was added to 1 μM TTR and fluorescence emission spectra were obtained with excitation of 320 nm. a) The resveratrol fluorescence intensity at 394 nm (open circles)and the tryptophan center of spectral mass (filled circles) are plotted as a function of urea concentration for each TTR variant (indicated in each panel).b) Percentage of tetramer as a function of urea concentration. c) Standard lines for each TTR variant (from 0.2 to 1.8 μM tetramer concentration) with a fixed resveratrol concentration (18 μM). The fluorescence intensity at 394 nm is plotted as a function of TTR tetramer concentration. The fluorescence data is fit by linear regression. Resveratrol fluorescence data were used to quantify the concentration of tetramers at increasing urea concentrations and estimate the percentage of tetramers shown in b.

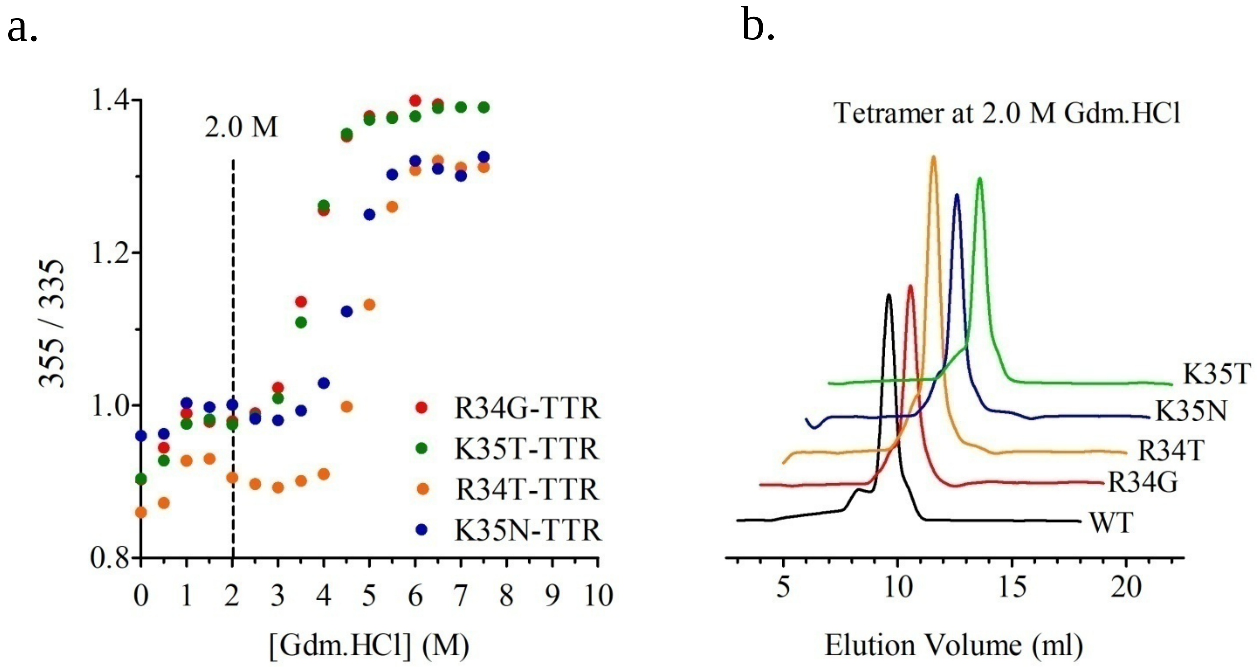

Figure S5. *Guanidinium chloride equilibrium denaturation of TTR disease associated variants and hydrodynamic behaviour of species populated at 2.0 M Gdm.HCl.*

(*a*) 1.5 μM of WT and pathogenic TTR variants were incubated for 96 h at 25 °C in 50 mM sodium phosphate (pH 7.0), 0.1 M KCl with increasing Gdm.HCl concentrations prior measurements of tryptophan fluorescence emission spectra. (b) Size exclusion chromatography in an anlytical Superdex 75 10/300 GL column (GE). The column was equilibrated in 50 mM sodium phosphate (pH 7.4), 0.1 M KCl and 2.0 M Gdm.HCl. The samples were incubated for 72 h in equilibration buffer at 25 °C and centrifuged at 12000 rpm for 15 min. prior injection.

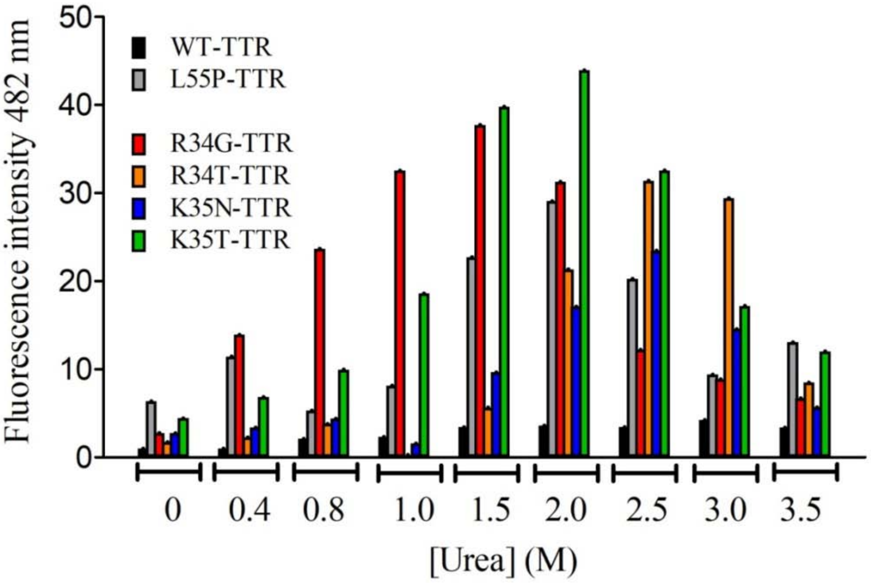

Figure S6. *Th-T binding of urea denaturation intermediates of TTR variants. Urea denaturation experiments of WT and TTR variants were performed at 15* µM as described in Figure S2, After tryptophan fluorescence measurements, 1.5 µM of the samples were incubated with 30 µM ThT in 100 mM Tris.HCl (pH 8.0), 0.1 M KCl and the fluorescence intensity was recorded at 482 nm (excitation 440 nm)

a.

b.

c.

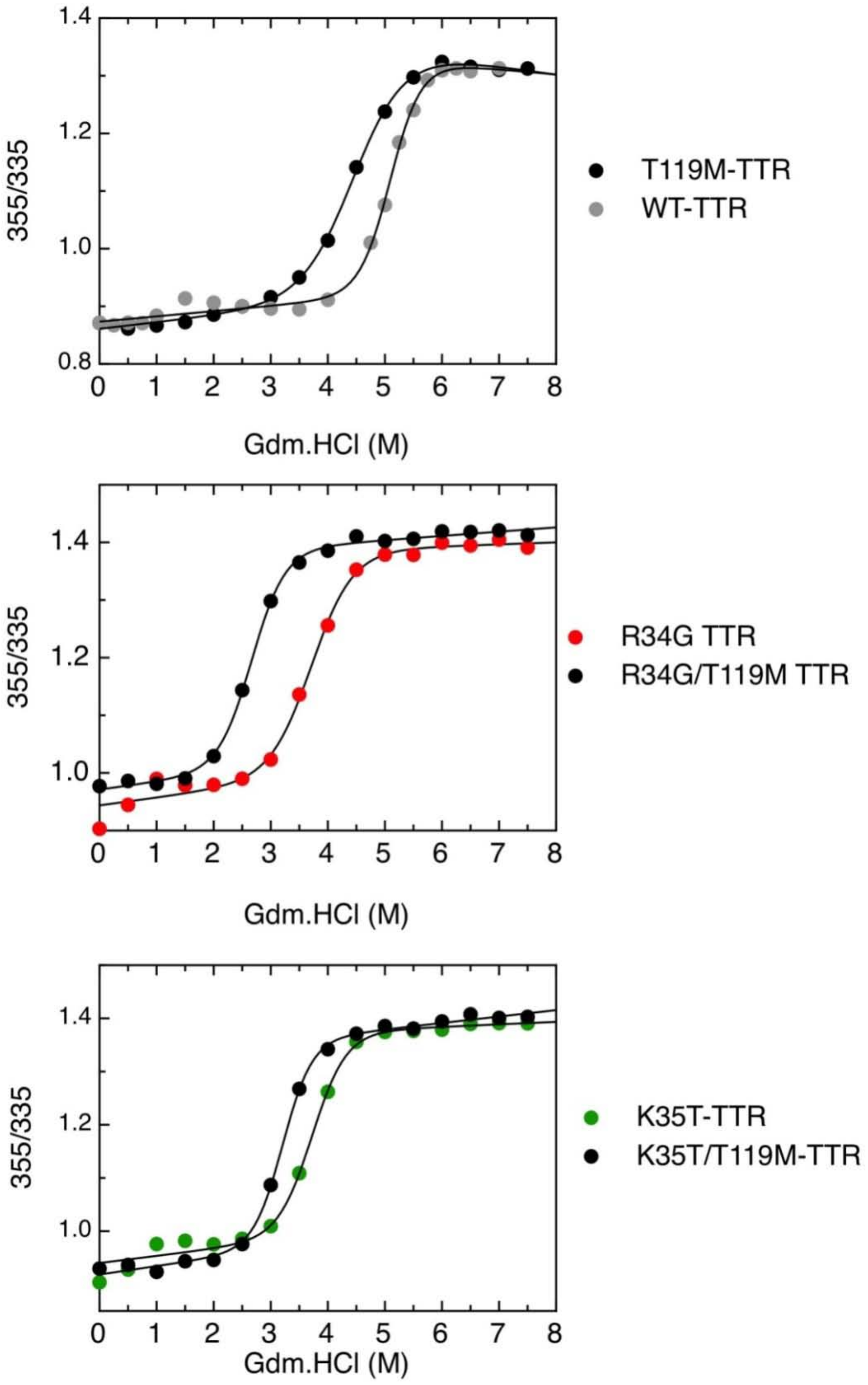

Figure S7. *Comparison of guanidinium chloride equilibrium denaturation experiments of TTR variants harboring T119M. (a)* 1.5 μM of TTR variants were incubated for 72 h at 25 °C in 50 mM sodium phosphate (pH 7.0), 0.1 M KCl with increasing Gdm.HCl concentrations prior fluorescence measurements. The fluorescence 355/335 ratio is plotted as a function of Gdm.HCl concentrations. Denaturation curves of: WT and T119M TTRs (a), R34G and R34G/T119M TTRs (b), K35T and K35T/T119M TTRs (c). Lines connecting data points assuming a two-state deanturation model are represented

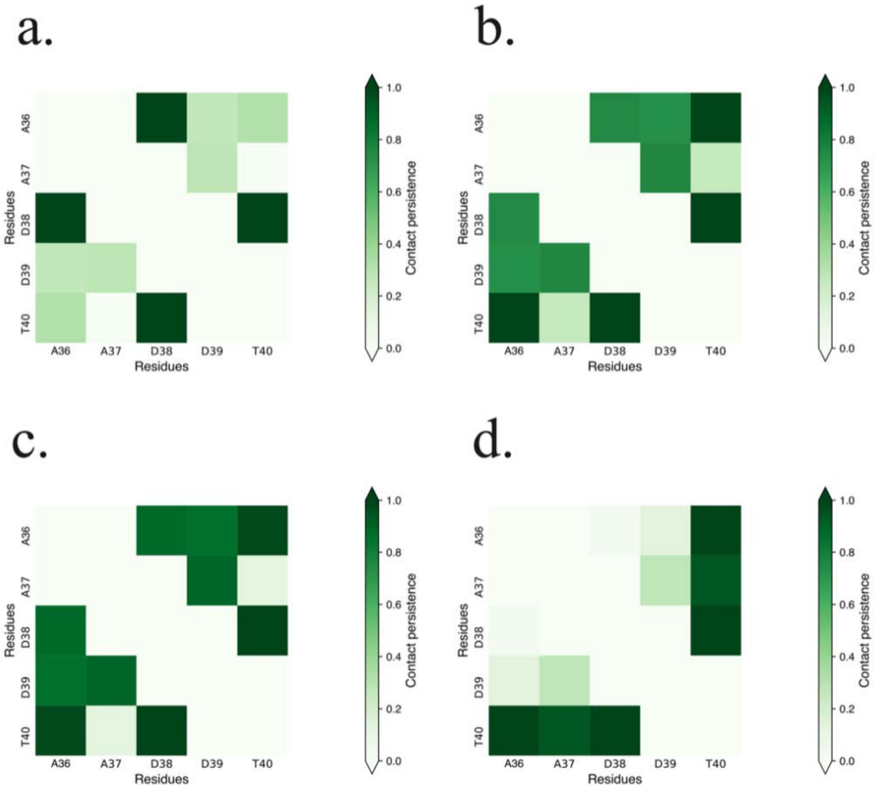

Figure S8. *Effect of the R34G and K35T mutations on the dynamics of the loop region 36-40.* Residue-residue contacts in the loop region 36-40 in the simulation of the R34G mutant, a) chain A and b) chain B, and in the simulation of the K35T mutant, c) chain A and d) chain B. Contacts are color-coded according to their persistence (number of frames of the simulations in which they are present over the total number of frames), with darker green cells representing more persistent contacts.

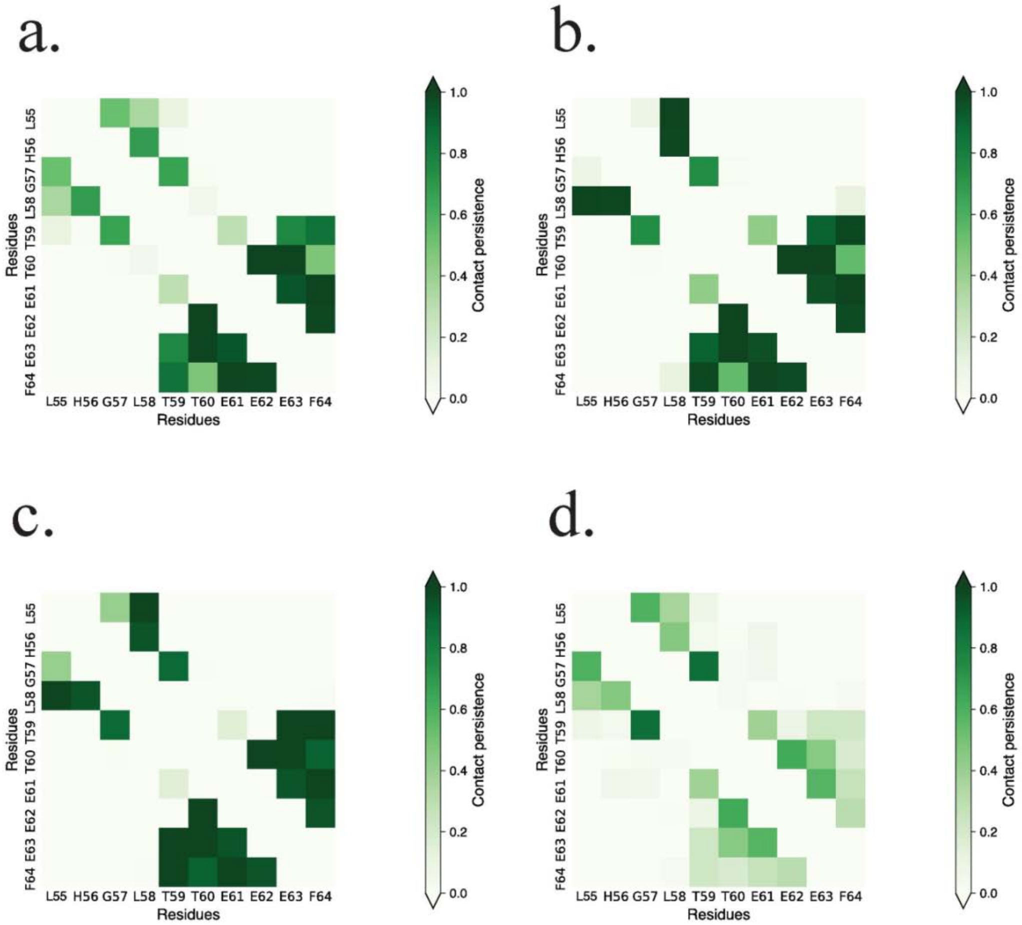

Figure S9. *Effect of the R34G and K35T mutations on the dynamics of the loop region 55-64.* Residue-residue contacts in the loop region 55-64 in the simulation of the R34G mutant, a) chain A and b) chain B, and in the simulation of the K35T mutant, c) chain A and d) chain B. Contacts are color-coded according to their persistence (number of frames of the simulations in which they are present over the total number of frames), with darker green cells representing more persistent contacts.

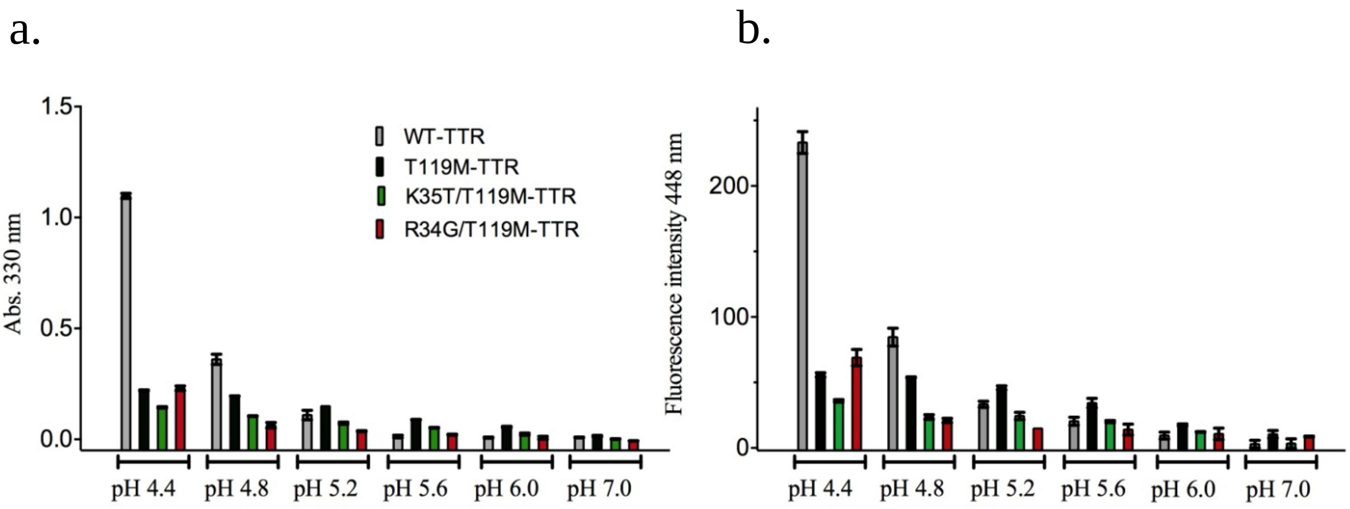

Figure S10. *Aggregation and fibril formation of TTR double mutants*.

3.5 μM of WT-TTR, T119M-TTR , R34G:T119M-TTR or K35T:T119M-TTR were incubated for 72 h at 37°C in 100 mM Tris.HCl, 50 mM MES, 50 mM sodium acetate and 0.1 M KCl, ranging from pHs 4.4 to 7.0. After incubation the samples were measured spectrophotometrically at 330 nm (a) and subjected to ThT binding assays (b).

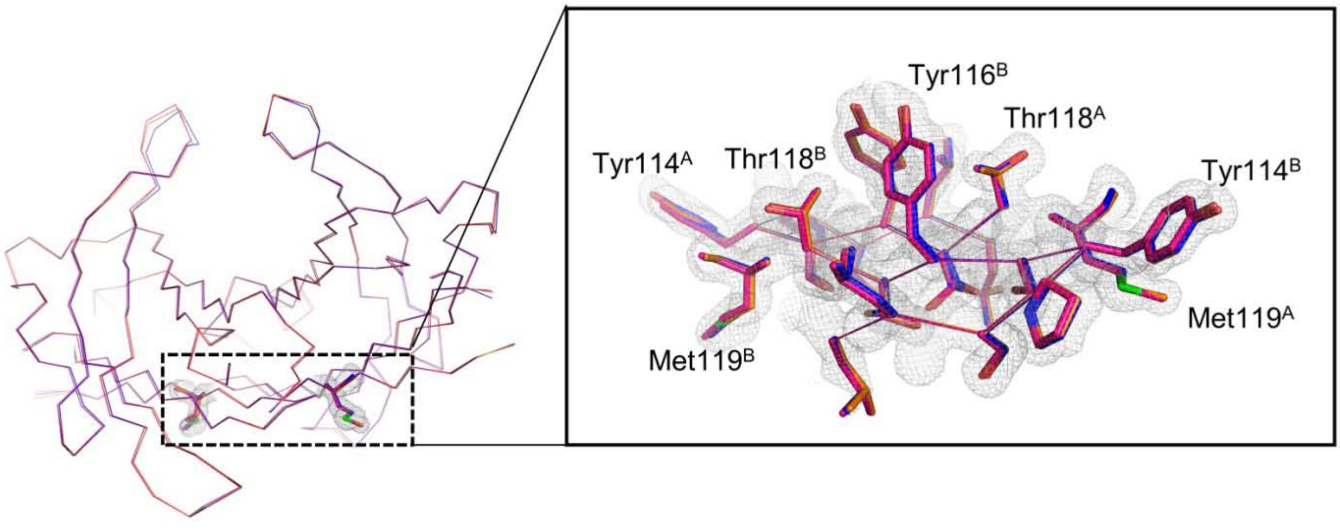

Figure S11 – *Cα superimposition of TTR variants at pH 5.5*. T119M (blue), K35T/T119M (orange), R34G/T119M (magenta). (PDB codes 6FXU, 6FZL, 6FWD). Met119 and surrounding residues are highlighted as sticks contoured the electron density map

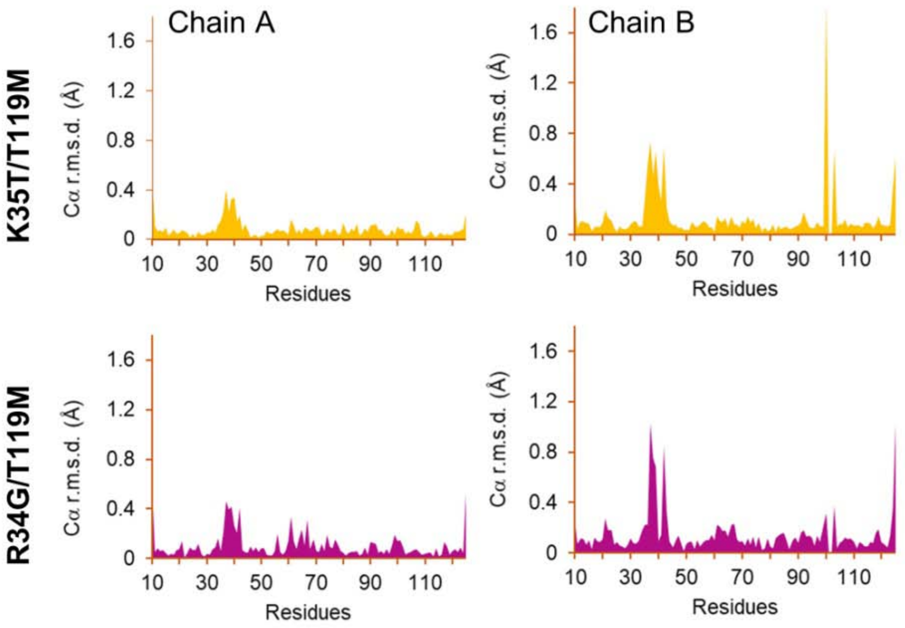

Figure S12. Plots of Cα R.M.S.D. for the chain A and B of K35T/T119M and R34G/T119M aligned to T119M-TTR at pH 5.5. Major differences are seen in the region of the BC-loops
